# Possible function of the second RecJ-like protein in stalled replication fork repair by interacting with Hef

**DOI:** 10.1038/s41598-017-17306-0

**Published:** 2017-12-05

**Authors:** Mariko Nagata, Sonoko Ishino, Takeshi Yamagami, Jan-Robert Simons, Tamotsu Kanai, Haruyuki Atomi, Yoshizumi Ishino

**Affiliations:** 1 Department of Bioscience and Biotechnology, Graduate School of Bioresource and Bioenvironmental Sciences, Kyushu University, Fukuoka, Japan; 2 Department of Synthetic Chemistry and Biological Chemistry, Graduate School of Engineering, Kyoto University, Kyoto, Japan

## Abstract

RecJ was originally identified in *Escherichia coli* and plays an important role in the DNA repair and recombination pathways. *Thermococcus kodakarensis*, a hyperthermophilic archaeon, has two RecJ-like nucleases. These proteins are designated as GAN (GINS-associated nuclease) and HAN (Hef-associated nuclease), based on the protein they interact with. GAN is probably a counterpart of Cdc45 in the eukaryotic CMG replicative helicase complex. HAN is considered mainly to function with Hef for restoration of the stalled replication fork. In this study, we characterized HAN to clarify its functions in *Thermococcus* cells. HAN showed single-strand specific 3′ to 5′ exonuclease activity, which was stimulated in the presence of Hef. A gene disruption analysis revealed that HAN was non-essential for viability, but the Δ*gan*Δ*han* double mutant did not grow under optimal conditions at 85 °C. This deficiency was not fully recovered by introducing the mutant *han* gene, encoding the nuclease-deficient HAN protein, back into the genome. These results suggest that the unstable replicative helicase complex without GAN performs ineffective fork progression, and thus the stalled fork repair system including HAN becomes more important. The nuclease activity of HAN is required for the function of this protein in *T. kodakarensis*.

## Introduction

All living organisms have replication systems to duplicate their genetic information, and have also evolved repair mechanisms to maintain genome integrity for their offspring and to prevent disorder of their cellular systems. The molecular mechanisms of DNA replication and repair have been actively studied since the discovery of the double-helix structure of DNA. A common feature is that these processes are performed by multiple protein complexes, called replisomes and repairsomes. Recent progress in the elucidation of the DNA replication mechanism has focused on the structure and function of the unwinding machinery, the unwindosome. In the eukaryotic DNA replication system, the Cdc45 protein forms a complex with the hexameric MCM, consisting of Mcm2-7, and the tetrameric GINS, consisting of Sld5, Psf1, Psf2, and Psf3 (CMG complex). Biochemical and structural analyses of the eukaryotic CMG complex are actively progressing^1–8^. Cdc45 and GINS perform structural roles to facilitate the formation of the active helicase complex by the catalytic MCM complex.

Archaea, the third domain of life, include very interesting organisms to study from molecular and evolutional biology aspects. Archaea have a unicellular ultrastructure without a nucleus, as observed for bacterial cells. However, the proteins involved in the genetic information processing pathways share strong similarities with those of Eukarya^9^. Actually, most of the DNA replication-related proteins in Archaea are homologues of the eukaryotic replication proteins, including ORC, Cdc6, GINS, MCM, RPA, PCNA, RFC, FEN1, in addition to the primase, DNA polymerase, and DNA ligase, which are obviously different from bacterial proteins^10,11^. Their similarities indicate that the DNA replication machineries of Archaea and Eukarya evolved from a common ancestor.

Regarding the replicative helicase, all archaeal genomes have at least one gene, and sometimes multiple genes, encoding an Mcm homolog^12^. Several archaeal Mcm proteins have been characterized *in vitro*, and the distinct DNA helicase activity of the MCM homohexamer has been shown *in vitro*, in contrast to the eukaryotic MCM. Furthermore, stimulation of the helicase activity by the interaction with GINS has been reported in several archaea^13–17^. It was particularly interesting to determine whether a counterpart of the eukaryotic Cdc45 exists in Archaea, because an obvious homolog had not been found in the archaeal genome. However, a detailed sequence analysis revealed that the eukaryotic Cdc45 protein possesses a DHH phosphodiesterase domain, which is also present in bacterial RecJs^18,19^. The bacterial RecJ protein has 5′–3′ ssDNA-specific exonuclease activity to function in DNA repair pathways, including homologous recombination, base excision repair, and mismatch repair^20–22^. In contrast, no nuclease activity has been detected for eukaryotic Cdc45 so far. A Cdc45/RecJ-like protein from the hyperthermophilic archaeon, *Thermococcus kodakarensis*, shows 5′–3′ single-stranded DNA (ssDNA)-specific exonuclease activity, as in the bacterial RecJs, and this nuclease activity is stimulated by GINS *in vitro* ^23^. This archaeal protein was designated as GAN, from GINS-associated nuclease. We demonstrated the GAN-GINS-MCM complex formation in *T. kodakarensis*, and determined the crystal structure of GAN complexed with the C-terminal B domain of the Gins51 subunit^24,25^. Furthermore, GAN-GINS stimulated the MCM helicase activity *in vitro*. Similarly, the formation of a complex of the Cdc45/RecJ-like protein with MCM and GINS from *Sulfolobus* species was reported. The association of the *Sulfolobus* Cdc45/RecJ with GINS and MCM robustly stimulated the helicase activity^26^. However, in the case of *Sulfolobus*, GINS had no effect on the helicase activity of MCM, as previously shown^27^, and Cdc45/RecJ alone did not show any detectable effect on the MCM helicase activity. In addition to these differences, we also described the CMG-like complex in *Thermoplasma acidophilum*, in which the Cdc45/RecJ-like protein is one of two homologs, RecJ1 and RecJ2. The CMG-like complex consists of Mcm, Gins51 (homotetramer in this archaeon), and RecJ2, which showed 3′–5′, but not 5′–3′, exonuclease activity *in vitro* ^28^. All of the reports suggested that the CMG-like complex, which includes Cdc45/RecJ, is the central component of the replicative helicase in Archaea, but the Cdc45/RecJ component is remarkably diversified.

The *T. kodakarensis* genome has two different genes encoding Cdc45/RecJ-like proteins. We found the second Cdc45/RecJ-like protein during our search for proteins interacting with the intrinsically disordered region (IDR) of the Hef (helicase-associated endonuclease for fork-structured DNA) protein, and designated this protein as HAN (Hef-associated nuclease)^29^. Hef was originally discovered in the hyperthermophilic archaeon, *Pyrococcus furiosus*. Biochemical characterization of the purified protein confirmed the specific affinity for branched DNA structures, including the replication fork. The helicase activity from the N-terminal domain was dramatically stimulated by fork-structured DNAs, and the endonuclease activity from the C-terminal domain specifically cleaved nicked, flapped, and fork-structured DNAs^30,31^. Genetic analyses using the *hef* mutant strain confirmed that Hef is involved in multiple repair processes, and its especially high sensitivity to mitomycin C (MMC) implied that Hef performs a critical function in DNA interstrand cross-link (ICL) repair^32^. These genetic and biochemical properties of Hef suggested that this protein actually works at stalled replication forks in *T. kodakarensis*, by the coordination of its helicase and endonuclease activities. Genetic and cytological analyses of the *hef* gene in *Haloferax volcanii* also revealed that Hef is involved in stalled replication fork repair^33,34^. The human ortholog of Hef (hHef) was identified as FANCM, and it is known that mutations in the *FANCM* gene are the cause of Fanconi anemia, a hereditary genetic disease^35,36^. Hef is now recognized as a protein involved in stalled replication fork repair. The biochemical properties of HAN have remained elusive, although our previous study showed its interaction with Hef ^29^. In this study, we biochemically characterized the highly purified HAN protein and isolated the *han* gene knock-out mutant of *T. kodakarensis*. Based on the biochemical properties and the phenotype of the mutant, we discuss the function of HAN in archaeal cells.

## Results

### HAN constitutes an independent cluster in the phylogenetic tree of RecJ family proteins

**Figure 1:**
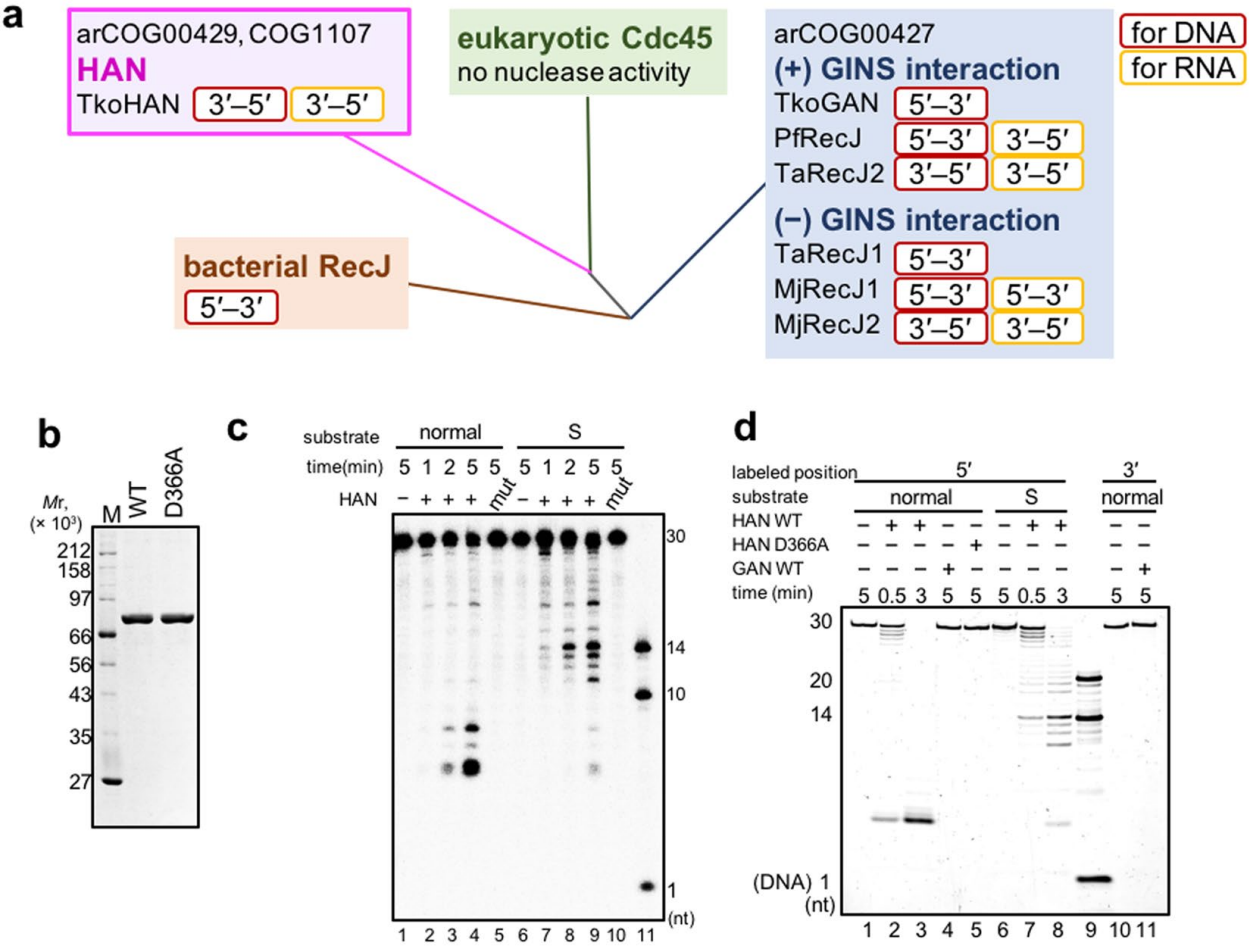
Characterization of the nuclease activity of HAN. (**a**) Schematic representation of the classification of the Cdc45/RecJ family members. Reported exonuclease activities and their directions are shown. TkoGAN, PfRecJ, TaRecJ1 and RecJ2, and MjRecJ1 and RecJ2 are RecJ-like proteins in *T. kodakarensis, P. furiosus, T. acidophilum*, and *M. jannaschii*, respectively. (**b**) Purified recombinant HAN-WT (2 μg) and HAN-D366A (2 μg) proteins were subjected to SDS -10% PAGE followed by CBB staining. Protein size markers were run in lane M, and their sizes are indicated on the left of the gels. (**c**) Detection of the cleavage direction of HAN for ssDNA. HAN (WT and D366A mutant) (5 nM each) was incubated with 5′-^32^P-labeled 5 nM ssDNA (dA30) at 70 °C for 1, 2, and 5 min. The 30-nt ssDNAs with or without four successive phosphorothioate modifications from the 10th to 14th bases were used for the substrates and indicated as “normal” (dA30, lanes 1–5) and “S” (dA30ssss14, lanes 6–10), respectively. Lane 11 shows the size marker DNAs (14, 10, and 1 nt, respectively). (**d**) HAN exhibited 3′ to 5′ exonuclease activity for RNA. GAN and HAN (WT and D366A) (5 nM each) were incubated with 5′- or 3′-FITC-labeled 10 nM RNA at 80 °C for 0.5, 3, and 5 min. HAN cleaved RNA processively (lanes 2 and 3). Substrates in these assays were 5′-FITC-labeled “normal” RNA (FITCrA30, lanes 1–5), 5′-FITC-labeled phosphorothioated (“S”) RNA (FITCrA30ssss14, lanes 6–8), and 3′-FITC-labeled RNA (rA30FITC, lanes 10–11). Products were subjected to 8 M urea-15% PAGE. Lane 9 shows 5′ FITC-labeled RNA (20 nt and 14nt) and DNA (1 nt) size markers.

RecJ family nucleases have been actively studied, as the predicted archaeal counterparts of Cdc45^23,25,27,28,37–39^. To understand their structure-function relationships, published RecJ-like proteins were picked up with their properties (referred above) and were schematically added to the phylogenetic tree, which were calculated by Makarova *et al*.^19^ (Fig. 1a). Bacterial RecJ has been regarded as a 5′ to 3′ exonuclease. However, no experimental data are available for the nuclease activity of eukaryotic Cdc45. The GAN ortholog is well conserved in many archaeal genomes (arCOG00427)^19^. However, it is interesting that a different group of proteins, which are not associated with GINS, is also included in this cluster. HAN, the second RecJ-like protein in *T. kodakarensis*, has typical DHH motifs, which are essential for the nuclease function in bacterial RecJs^40–42^. The characteristic feature is that HAN lacks motif V and the CID (CMG-Interaction domain)-like domain, which are unique to the GAN-family^24,28,39^ (Supplementary Fig. S1). This C-terminal region containing the DHH motifs is fused to three nucleic acid (NA)-binding domains, a DnaJ-like zinc finger (ZF) domain and two distinct OB-fold domains, at its N-terminal region. Based on these structural features, we expected that HAN possesses a unique exonuclease activity. Interestingly, HAN is conserved only in the euryarchaeal phylum, except for *Thermoplasmatales*, in Archaea (Supplementary Fig. S2).

### Biochemical characterization of the nuclease activity of HAN

We first investigated the nuclease activity of the highly purified HAN (Fig. 1b). As shown in Fig. 1c, the 5′-^32^P-labeled ssDNA substrate was shortened during the reaction. The substrate DNA became shorter than 10 nt, but was not degraded to single nucleotides (lanes 1–4, “normal” DNA). When HAN was reacted with the substrate DNA with four successive phosphorothioate modifications, the degradation was inhibited at the modification sites to produce 11–14 nt DNAs (lanes 6–9, “S” DNA). These results indicate that HAN cleaved ssDNA in the 3′ to 5′ direction. We also tested the nuclease activity of HAN using 3′-FITC-labeled DNA with or without phosphorothioated modification at 3′ terminus (Supplementary Fig. S3a), and found a product shorter than 1 nt from only DNA without phosphorothioated modification (lane 4). The DNA with phosphorothioated modification was not cleaved. The reaction rate of HAN nuclease was high at 70 °C (Supplementary Fig. S3b), although it was slow down to show the products as a ladder pattern at 50 °C (Supplementary Fig. S3c, lanes 3 and 4). To investigate whether the HAN reaction is processive, excess amounts (10- and 100-times) of non-labeled substrate DNAs were used to protect HAN, which fell down from the substrate DNA, from reloading. These experiments also showed only the very short product (Fig. S3d), and therefore, HAN should be a highly processive exonuclease. HAN cleaved 3′-overhanged dsDNA, but not blunt ended dsDNA and 5′-overhanged dsDNA (Supplementary Fig. S3c, lanes 6–12). The cleavage of the 3′-overhanged DNA stopped at about 10 nt from the double-stranded region, supporting the conclusion that HAN specifically cleaves ssDNA in the 3′ to 5′ direction. HAN did not degrade circular ssDNA and dsDNA (Supplementary Fig. S3e), indicating that HAN lacks endonuclease activity. In addition, HAN cleaved RNA with the same or slightly better efficiency (Supplementary Fig. S3f). HAN cleaved 5′-FITC-labeled RNA and generated <14 nt products (Fig. 1d, lanes 2 and 3), but not RNA phosphorothioated at the same region as the DNA substrates (Fig. 1d lanes 7 and 8). GAN did not cleave RNA from either the 5′ or 3′ end (lanes 4 and 11), as previously observed^23^. As a control, the DNase activity of the GAN protein used for this experiment was shown in Fig. S3g. The nuclease activity of HAN was clearly dependent on manganese, and magnesium could also be used with much less efficiency. Calcium and zinc were not utilized by HAN (Supplementary Fig. S4). The metal-binding residue in motif I was predicted from the bacterial RecJ and GAN, and the alanine substitution mutant, HAN-D366A, was prepared (Fig. 1b) and used for the nuclease assay. This mutation almost completely inactivated HAN (Fig. 1c, lanes 5 and 10; Fig. 1d, lane 5). The loss of the exonuclease activity in HAN-D366A is consistent with the results of equivalent mutations in bacterial RecJ and archaeal RecJ-like proteins^25,38,40,43^.

### Specific interactions of HAN

**Figure 2:**
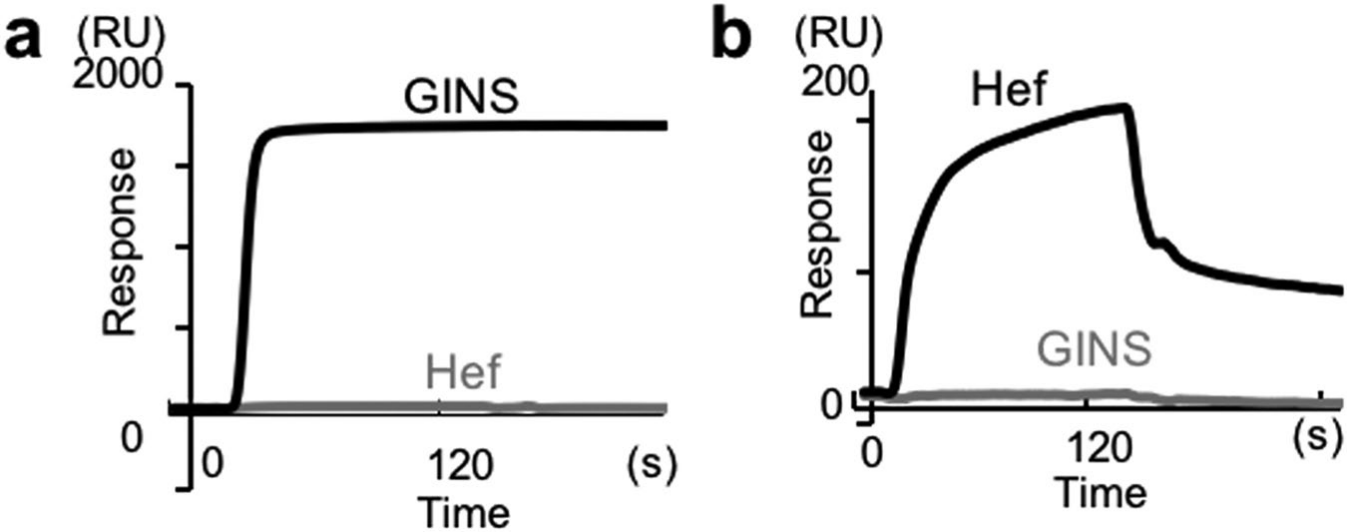
Specific interactions of GAN - GINS and HAN - Hef. GINS and Hef-IDR proteins were loaded on a GAN-bound chip (**a**) and a HAN-bound chip (**b**). The loading proteins are indicated on the sensorgrams. The sensorgram of GINS–GAN reported in Oyama *et al*.^24^ was also shown on panel (a) for comparison.

We previously reported the physical interactions of GAN–GINS and HAN–Hef, detected by SPR-analyses^24,25,29^. We tested whether GAN and HAN could substitute for each other by SPR analyses, using the highly purified GAN, GINS, HAN, and Hef (IDR) proteins. GINS bound the GAN-immobilized sensorchip, but Hef-IDR did not (Fig. 2a). Hef-IDR bound the HAN-immobilized sensorchip, but GINS did not (Fig. 2b). These results clearly showed that the interactions of GAN–GINS and HAN–Hef are specific, and there is no complementarity between the two RecJ-like proteins.

### Co-localization of HAN and Hef in *T. kodakarensis*

**Figure 3:**
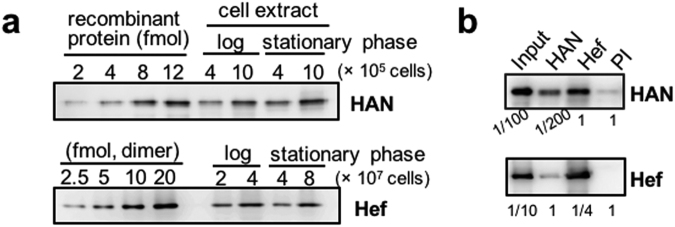
Detection of HAN and Hef and their co-localization in *T. kodakarensis* cells. (**a**) Estimation of the intracellular concentrations of HAN and Hef in *T. kodakarensis* cells. The loading amounts and detection conditions were depending on the activities of the antibodies. (**b**) Immunoprecipitation of HAN and Hef from the *T. kodakarensis* cell extract. The immunocomplexes were captured individually with each antiserum from the whole cell extract (as shown at the top), and were subjected to SDS-10% PAGE followed by western blot analyses using each antiserum (shown on the right side). The whole cell extracts without immunoprecipitation (Input) and those precipitated after a treatment with preimmune serum (PI) were also loaded as positive and negative controls, respectively. The relative loading amounts are indicated at the bottom of the panels.

The intracellular amounts of HAN and Hef in exponentially- and stationary-phase *T. kodakarensis* cells were measured by western blot analyses, using anti-HAN and anti-Hef antisera. The amounts of the HAN and Hef proteins were calculated to be 10,000–15,000 molecules/cell (as the monomer) and 50–200 molecules/cell (as the dimer), respectively, based on a comparison of the band intensities from the cell extract and serial dilutions of each purified protein (Fig. 3a). The intracellular concentrations of HAN and Hef should be about 5 µM and 0.1 µM, respectively, in a *T. kodakarensis* cell, calculated from a spherical cell with a 1 μm diameter. HAN is over 10-fold more abundant than Hef and GAN^25^. To investigate whether the HAN and Hef proteins could form a complex in *T. kodakarensis* cells, an immunoprecipitation experiment was performed. The immunocomplexes were captured with either anti-HAN or anti-Hef antibodies from the whole cell extracts. The immunocomplexes were subjected to SDS-PAGE, followed by western blot analyses using each antibody. As shown in Fig. 3b, HAN and Hef co-precipitated with each of the antibodies, suggesting that HAN and Hef exist in the same complex in *T. kodakarensis* cells. However, the stoichiometric ratio of the two proteins is clearly different as described above, and therefore, many free HAN molecules should exist to share some other functions in the cells.

### Effects of HAN and Hef on stalled fork DNA

Synthetic fork-structured DNAs were used for the nuclease assays of Hef and HAN. To detect the precise cleavage site, the template strand for leading synthesis and the nascent leading strand were labeled with FITC at the 3′-terminus and Cy5 at the 5′-terminus, respectively (Supplementary Fig. S5). The splayed arm DNA (sub1) was not cleaved by Hef, as reported previously. The fork DNA without lagging strand synthesis (sub2) was slightly cleaved at the nascent leading strand on the template. However, the fork DNA with no gap at the nascent lagging strand (sub3) was clearly cleaved at the template for leading strand synthesis. These results confirmed that the fork-structured DNA without a lagging strand gap is a preferable substrate for Hef, and the single-stranded nascent DNA strand, produced by the fork regression, is not cleaved. Furthermore, we also confirmed that Hef cleaves the template strand for leading strand synthesis with the same efficiency in either the presence or absence of the ssDNA region corresponding to the nascent leading strand produced by fork regression (Supplementary Fig. S6).

**Figure 4:**
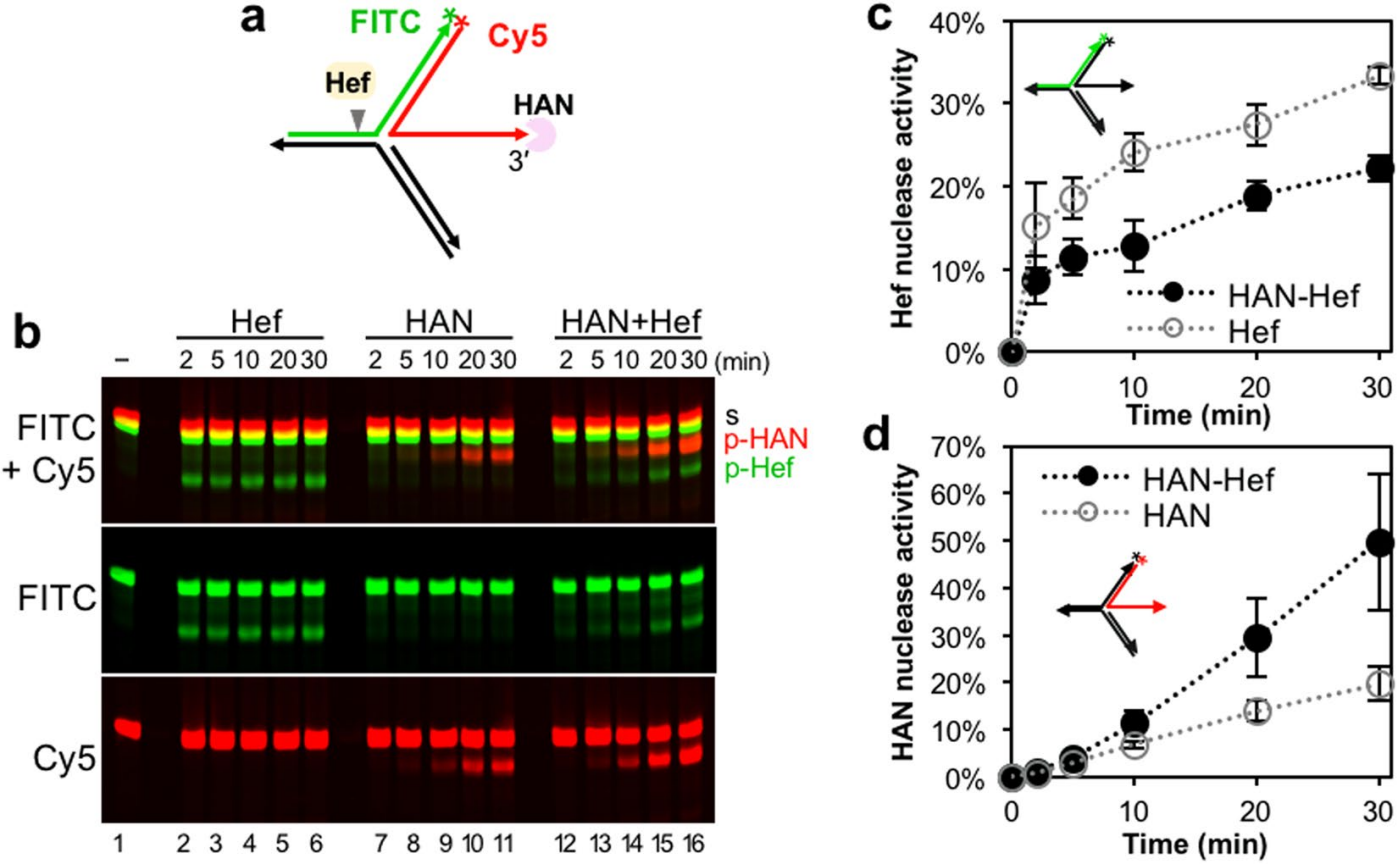
Mutual regulation of the nuclease activities of Hef and HAN. (**a**) The structure of the substrate is shown with the sites labeled by FITC and Cy5. The site cleaved by Hef is indicated by arrowhead. (**b**) The effects of HAN and Hef on each other on the fork-structured DNA. Hef (5 nM as the dimer) and HAN (100 nM) were incubated with 50 nM of each substrate at 50 °C for 2, 5, 10, 20, and 30 min. The reaction products were analyzed by 8 M urea-15% PAGE. The band assignments are indicated on the side of the panel: s, substrates; p, cleaved products by HAN (p-HAN, Cy5-labeled DNA) and Hef (p-Hef, FITC-labeled DNA). (**c**) Quantification of Hef nuclease activity in the presence or absence of HAN. The FITC-labeled cleavage products generated by Hef in panel b were quantified and represented as percentage of the total amount of substrate DNA. The SEM was calculated from three independent experiments. (**d**) Quantification of HAN nuclease activity in the presence or absence of Hef. The Cy5-labeled products generated by HAN in panel b were quantified and plotted. The SEM was calculated from three independent experiments.

Based on the interaction with Hef, we assumed that HAN could work at stalled fork DNA, and possibly cleaved the exposed-ssDNA produced by regression of the nascent leading strand. We tested the effects of HAN and Hef on the regressed fork DNA containing a single-stranded nascent leading strand, as shown in Fig. 4a (corresponding to sub3 in Supplementary Fig. S5). We incubated 100 nM HAN and 5 nM Hef (as the dimer) with this fluorescently-labeled regressed fork DNA with 100 mM Mg^2+^ and 0.1 mM Mn^2+^, according to their intracellular concentrations^25^. As shown in Fig. 4b, the FITC-labeled products corresponding to the Hef reaction were decreased in the presence of HAN, as compared to those by Hef alone. In contrast, the Cy5-labeled products by HAN were clearly increased in the presence of Hef, as compared with those in the absence of Hef. These inhibitions and stimulations were observed repeatedly, and the quantitative results are shown in Fig. 4c and d. When we analyzed the sites cleaved by Hef, the nicking site in the template strand for leading synthesis was the same in the presence and absence of HAN. The hydrolysis from the 3′-terminus of the strand corresponding to the nascent leading strand by HAN was stopped at 11–13 nt from the junction (Supplementary Fig. S7), which is longer than that of the products from the simple ssDNA (Fig. 1c and Supplementary Fig. S3), probably because the fork-structure sterically hindered the grasping of the single-stranded DNA region.

### HAN is not essential for viability, but the disruption caused a feeble phenotype in *T. kodakarensis*

**Figure 5:**
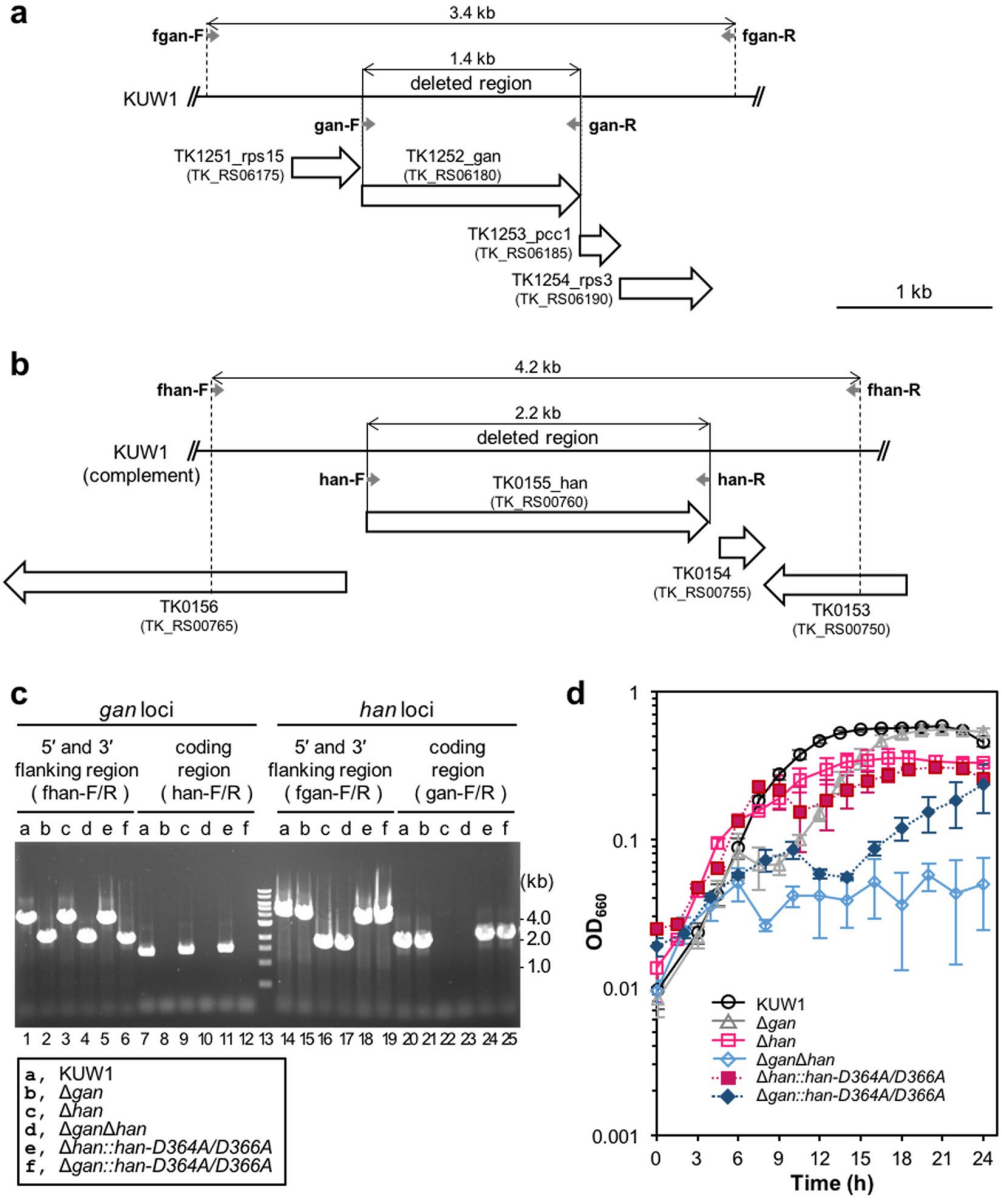
Disruption of the *han* gene slightly affect the growth of *T. kodakarensis*. (**a**,**b**) Schematic map of the gene organizations at the *gan* (a) and *han* (b) loci in the *T. kodakarensis* genome. (**c**) The confirmation of the gene disruptions and revertants by PCR analyses of the *gan* and *han* loci. *T. kodakarensis* parental strain (KUW1), Δ*gan*, Δ*han*, Δ*gan*Δ*han*, Δ*han::han-D364A/D366A*, and Δ*gan::han-D364A/D366A* strains are indicated at the top of the panel as a, b, c, d, e, and f, respectively. The length of the amplified fragments expected either before or after recombination are 3400 bp (lanes 1, 3, and 5) or 1989 bp (lanes 2, 4, and 6) for the 5′ and 3′ flanking regions of the *gan* locus by using the fgan-F/R primer set, 1452 bp (lanes 7, 9, and 11) or 0 bp (lanes 8, 10, and 12) for the coding regions of the *gan* locus by using the gan-F/R primer set, respectively, and 4194 bp (lanes 14, 15, 18, and 19) or 1968 bp (lanes 16 and 17) for the 5′ and 3′ flanking regions of the *han* locus by using the fhan-F/R primer set, 2243 bp (lanes 20, 21, 24, and 25) or 0 bp (lanes 22 and 23) for the coding regions of the *han* locus by using the han-F/R primer set, respectively. DNA size markers were run in lane 13, and their sizes (kb) are indicated on the right of the panel. (**d**) Growth properties of the *T. kodakarensis* mutant strains in ASW-YT-Pr at 85 °C. Symbols are indicated at the bottom of the panel. Growth was monitored by turbidity at 660 nm (OD_660_). Each strain was examined at least thrice, and error bars represent SEM.

To analyze the functions of HAN in *T. kodakarensis* cells, we tried to disrupt the *han* gene on the genome, and isolated the *han* deletion mutant strain. We previously reported the Δ*gan* mutant, and in this study, the *gan* and *han* (Δ*gan*Δ*han*) deletion mutant was also isolated successfully. PCR amplification of the *gan* and *han* regions from each mutant strain produced DNA products with the predicted size, corresponding to the lengths of the 5′ and 3′ flanking regions of the targeted loci without the GAN- or HAN-coding regions (Fig. 5a–c). Therefore, both GAN and HAN are not essential for *T. kodakarensis* viability, at least under the normal growth conditions employed for selection (growth in ASW-YT-S^0^ medium at 85 °C). No effect of the *gan* gene deletion on neighboring gene expressions was carefully confirmed in our previous report, because other ORFs exist in the immediate vicinities of the *gan* gene^25^. There are some intergenic regions both up and downstreams of the *han* gene. (Fig. S8a), and we predicted no effect of the deletion of just the coding region of HAN on the vicinities in this study. We measured the growth properties of the mutants in ASW-YT-Pr medium at 85 °C, and detected a slight growth defect of Δ*han* as compared with the parental strain, KUW1, and the growth-plateau of Δ*han* seemed to be lower than those of KUW1 and Δ*gan*. We also found that the Δ*gan*Δ*han* mutant cells did not grow in ASW-YT-Pr medium at 85 °C (Fig. 5b). The effect of the cell morphology on turbidity of the cell culture was checked by microscopic observations of the growing cells with different conditions. The cells were always observed as a typical cocci after growing at 93 °C or irradiated by 100 and 500 J/m^2^ of UV, and were not different from the cells grown in the normal cultivation condition. The growth defects described above were reproducibly observed more than three times, with three independently isolated strains. The Δ*gan*, Δ*han*, and Δ*gan*Δ*han* mutants, as well as the parental KUW1 cells, grew normally in ASW-YT-Pr medium at 70 °C (Supplementary Fig. S8). We tested whether the growth defects of Δ*han* and Δ*gan*Δ*han* are derived from the loss of the HAN nuclease activity, by inserting the nuclease-deficient *han* gene (*han-D364A/D366A*) into the Δ*han* and Δ*gan* strains, Δ*han::han-D364A/D366A* and Δ*gan*:*:han-D364A/D366A*. The growth of the Δ*han::han-D364A/D366A* cells did not recover to that of KUW1, and the Δ*gan*:*:han-D364A/D366A* cells showed additional growth defects as compared with those of Δ*gan* (Fig. 5b). These observations indicate that the nuclease activity of HAN is important for its functions in the *T. kodakarensis* cells. However, Δ*gan*:*:han-D364A/D366A* did not show the severe temperature-sensitive phenotype at 85 °C, in contrast to the Δ*gan*Δ*han* cells, suggesting that HAN functions not only as a nuclease but also as an important component for survival at high temperature.

### Damage sensitivity of the Δ*han mutant*

**Figure 6:**
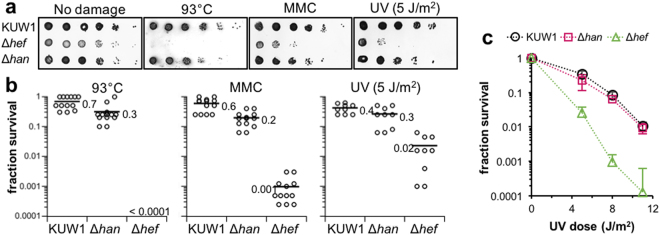
Sensitivities of the Δ*han* mutant to DNA-damaging treatments. (**a**) Drop dilution assays for the sensitivities of parental strain (KUW1), Δ*han*, and Δ*hef* cells to 93 °C, MMC, and UV-C. The serially diluted *T. kodakarensis* cells (5 × 10^5^, 5 × 10^4^, 5 × 10^3^, 5 × 10^2^, 5 × 10^1^, and 5 × 10^0^ cells from left to right) were spotted on the plates before the treatment, as described in the Materials and Methods. The plates were then incubated anaerobically at 85 °C [“No damage”, “MMC”, and “UV (5 J/m^2^)”] and 93 °C (“93 °C”). (**b**) Quantifications of the rates of surviving colonies from (**a**) were plotted from more than nine independent experiments using Kaleida Graph 4.5 (Synergy Software). The value of each bar depicts a mean determined from calculated plots. (**c**) The survival fraction curves (treated with 5, 8, and 11 J/m^2^ UV-C dose) of different strains were plotted. Each strain was examined at least thrice, and error bars represent SEM. Only the plus error bars are indicated for the Δ*hef* strain. Symbols are indicated on the top of the panels.

Since our previous analyses showed that the Δ*hef* mutant was highly sensitive to MMC, Hef may perform a critical function in stalled fork repair, especially those caused by ICLs^32^. We examined whether the Δ*han* cells displayed increased sensitivity to DNA damaging agents, as HAN may function with Hef in a DNA repair pathway. We monitored how the deletion of the *han* gene impacted the cell viability after DNA-damaging treatments, by a drop dilution assay (Fig. 6a). Under normal conditions without any DNA-damaging treatment, the Δ*han* mutant cells exhibited growth comparable to the parental KUW1 and the Δ*hef* mutant. To investigate the effects of damage induction on the growth of the Δ*han* and Δ*hef* mutants, their viabilities were compared at a higher temperature (93 °C), in the presence of MMC, and after UV irradiation. The growth defect of the Δ*hef* cells under these damage-inducing conditions was remarkable. The Δ*hef* cells could not form colonies upon cultivation at 93 °C for 24 h, as observed for the Δ*gan* cells previously^25^. The sensitivity of the Δ*han* cells to the high temperature increased slightly as compared with the parental KUW1 cells (Fig. 6a), and the viability was quantified by counting the survival rates of the cells (Fig. 6b). The Δ*hef* cells showed high sensitivity for MMC, as previously reported in *T. kodakarensis* and *H. volcanii* ^32,44^. The Δ*han* cells also showed some sensitivity to MMC, but less as compared to the Δ*hef* cells (Fig. 6a,b). We further examined the UV-sensitivity of the Δ*han* mutant. The Δ*hef* cells showed high sensitivity to 5 J/m^2^ of UV irradiation, consistent with our previous observation. However, the Δ*han* cells were obviously more resistant to this dose of UV irradiation, although they were slightly more sensitive than the parental KUW1 cells (Fig. 6a,b). This sensitivity of the Δ*han* cells was in the limits of error at higher doses (8, 11 J/m^2^) of UV irradiation, and the mean sensitivities of the parental strain and the Δ*han* cells were about the same (Fig. 6c).

## Discussion

We have demonstrated that *T. kodakarensis* HAN, the second RecJ-family protein, actually possesses 3′ to 5′ exonuclease activity for ssDNA and RNA, in contrast to bacterial RecJ, a 5′ to 3′ exonuclease. The GAN protein, identified as the first RecJ-like protein in *T. kodakarensis*, has 5′ to 3′ exonuclease activity that is stimulated by GINS, a component of the replicative helicase complex. We reported recently that the GAN nuclease lacks activity *in vitro* in the presence of 100 mM Mg^2+^ and 0.1 mM Mn^2+^, which are the intracellular concentrations in *T. kodakarensis* cells. In addition, the growth defect of the Δ*gan* mutant cells was complemented by the introduction of the nuclease-deficient mutant *gan* gene. From these results, we proposed that GAN works as a component of the replicative helicase and fulfills a structural role, similar to the case of eukaryotic Cdc45 in the CMG complex, even though GAN clearly has 5′ to 3′ exonuclease activity *in vitro* ^23–25^. In contrast to GAN, in this study we showed that HAN possesses 3′ to 5′ exonuclease activity under conditions with 100 mM Mg^2+^ and 0.1 mM Mn^2+^. Furthermore, the nuclease-deficient mutant *han* gene did not fully recover the temperature-sensitive phenotype of the Δ*gan*Δ*han* mutant cells. Therefore, the 3′ to 5′ exonuclease activity is actually important for HAN to function in *T. kodakarensis* cells.

**Figure 7:**
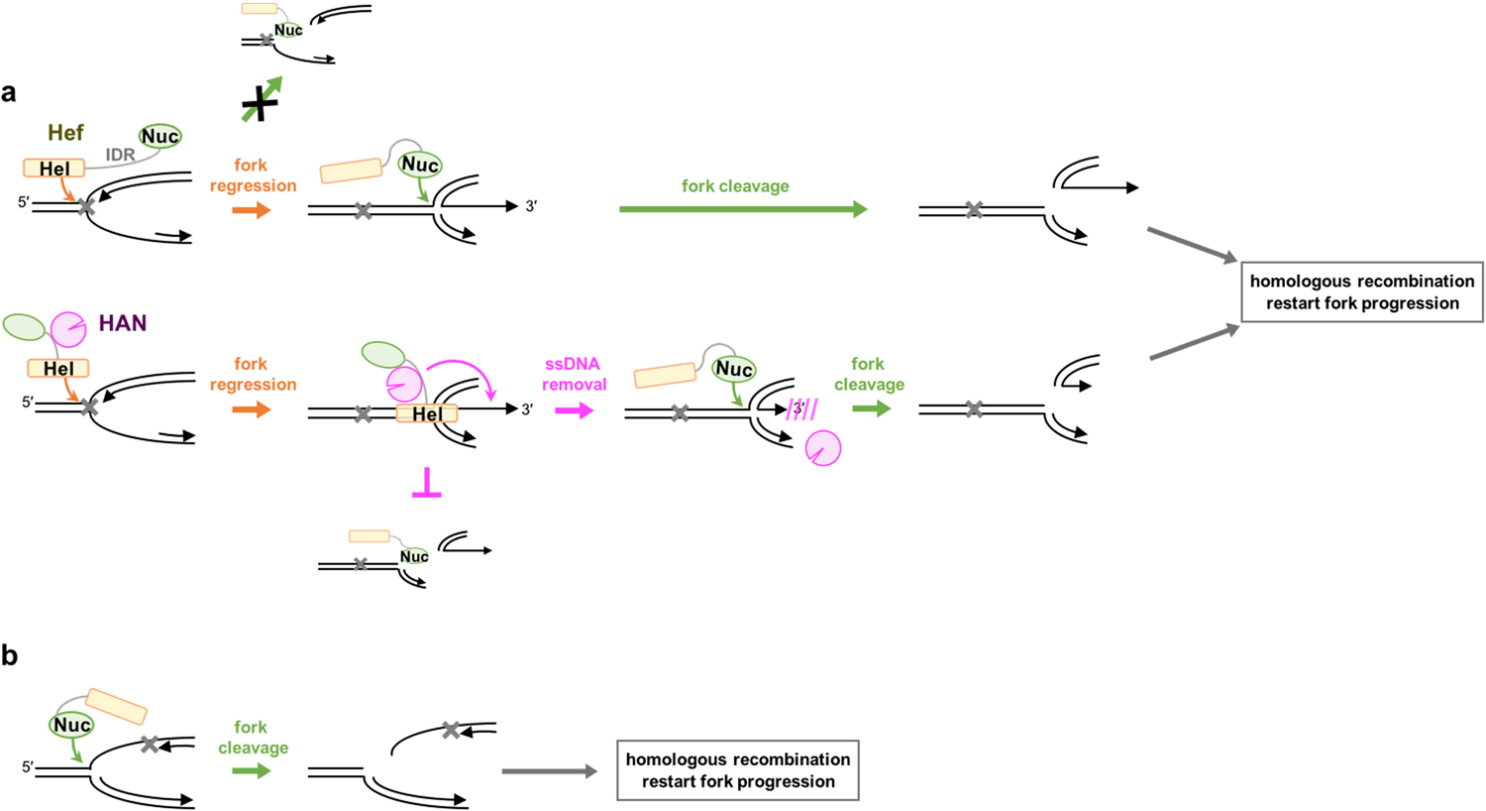
Proposed model of the cooperative function of HAN and Hef for stalled fork restart. The helicase and nuclease domains of Hef are drawn separately (for simplicity, Hef is drawn as monomer here). HAN works especially at the arrested fork by the delay of lagging strand synthesis (**a**), for which the helicase domain preferentially binds and unwinds the nascent leading strand to induce fork regression. In this case, HAN binds to the ID region of Hef. For the fork arrested by the delay of leading strand synthesis (**b**), the nuclease domain of Hef binds the fork junction and breaks the fork. See the text for more details.

HAN specifically interacts with Hef, which is probably involved in stalled replication fork repair, and therefore, the functional relationships between the two proteins have been predicted. We showed here that the 3′ to 5′ exonuclease activity of HAN was stimulated and the endonuclease activity of Hef was inhibited in the presence of both proteins *in vitro*. Based on these results, we propose a model in which Hef and HAN cooperatively work at the stalled replication fork (Fig. 7). The basic properties of Hef revealed that the endonuclease, which cleaves the junction of fork-structured DNA at the strand corresponding to the template for leading synthesis, does not work for a fork-structure containing a single-stranded gap on the lagging side (which happens when the lagging strand synthesis stops ahead and only leading strand synthesis proceeds). In this case, the helicase activity of Hef can unwind the nascent leading strand to promote the fork regression. The helicase domain and the nuclease domain are clearly separated and connected by an IDR in the Hef protein (schematically drawn in Fig. 7). Due to steric hindrance, these two domains cannot simultaneously access the junction site of the fork DNA. We predict that the HAN-bound Hef accesses the fork junction predominantly with the helicase domain. Then, the nascent leading strand is dissociated from the template to become a single strand with an exposed 3′-terminus, which is a preferable substrate for HAN. When the fork structured DNA is converted to the gap-less form in the lagging strand, the domain exchange from helicase to nuclease occurs. In the case of the stalled fork with a single-strand gap in the leading strand, the nuclease domain binds to the junction and cleaves the template DNA, as we observed previously. Notably, HAN may serve to determine which domain (helicase or nuclease) in Hef preferentially binds the stalled fork junction.

The effects of the *han* deletion mutation on the growth of *T. kodakarensis* cells were not serious under the normal growth conditions. Some 3′ to 5′ exonuclease activities derived from other proteins, such as DNA polymerase B and D^11^, and Exonuclease I^45^, may function as substitutes in the absence of HAN. However, the GAN and HAN double mutant (Δ*gan*Δ*han*) exhibited a temperature-sensitive phenotype at 85 °C. We previously reported that the Δ*gan* mutant showed a temperature-sensitive phenotype at 93 °C, but was normal at 85 °C^25^. We assume that the replicative helicase complex without GAN becomes unstable for sufficient progression at higher temperatures, and the replication fork tends to stop, as compared with the wild type. In this situation, the effect of the HAN deficiency becomes higher in the Δ*gan*Δ*han* mutant. We performed a targeted insertion of the nuclease-defective *han* gene (*han-D364A/D366A*) into the Δ*gan* strain. However, the *han-D364A/D366A* gene did not fully complement the temperature-sensitivity of the Δ*gan*Δ*han* mutant, in contrast to the case of GAN, in which the growth defects of the Δ*gan* strain were fully recovered by the introduction of the nuclease-deficient *gan* gene (*gan-D34A/D36A*) into the genome^25^. These results indicate that HAN works as a nuclease in *T. kodakarensis* cells, in contrast to GAN, which probably lacks nuclease activity in the cells. The partial complementation of the growth defect of the Δ*gan*Δ*han* mutant by the nuclease-deficient HAN may be due to the fact that HAN maintains the function to select the helicase domain in Hef to bind the stalled fork DNA, even though its nuclease active site is disrupted. There are 50–300 times more HAN molecules than Hef in *T. kodakarensis* cells, and it is possible that HAN plays another role independently from Hef. HAN works on both RNA and DNA, and it may also be involved in RNA metabolism. Screening for additional interacting partners of HAN in *T. kodakarensis* cells will be useful to clarify this issue.

Hef was originally identified in *P. furiosus*, which belongs to the euryarchaeal phylum in Archaea. The phylogenetic relationships of the RecJ family proteins revealed that the HAN family is separated from other groups, including archaeal GAN-like proteins, eukaryotic Cdc45s, and bacterial RecJs (Fig. 1a). Interestingly, the HAN family is conserved only in Euryarchaeota that have the Hef protein^29^ (Supplementary Fig. S2). These distributions support the cooperative functions of Hef and HAN in a euryarchaeal-specific repair pathway. Given their specific habitat, the euryarchaeal organisms may have developed a unique repair pathway with HAN and Hef for maintaining their genetic information. The homologous recombination process could follow the Hef-mediated fork disruption. Polyploidy may be a common trait of euryarchaeal cells^46^, and the copy number of chromosomal DNA in *T. kodakarensis* is 7–19 copies per cell^47^. The multiple copies of DNA would be advantageous for efficient homologous recombinational repair in euryarchaeal cells.

The domain organizations are clearly different between the GAN and HAN proteins. This difference is probably related to the specific properties of each protein. For example, the cleavage activity of GAN works only for ssDNA, starting from the 5′-end and proceeding to the 3′-end of the DNA strand. In contrast, HAN works on both ssDNA and RNA, and the cleavage starts from the 3′-end and stops at about <10 nt from the 5′-end. HAN probably needs to grasp about 10 nucleotides of the DNA strand to express its nuclease activity. The DnaJ-like ZF domain and two OB-fold domains (OB1 and OB2), found in the N-terminal region, are unique features of the HAN protein, although some of them lack ZF (Supplementary Fig. S2). These domains probably work for the DNA binding and confer the specificity. The structures and functions of the GAN family are more complicated. The domain organizations of the proteins in this family are unified, as shown in Supplementary Fig. S1. However, some are not associated with GINS (therefore, they are actually not GAN). RecJ1 from *T. acidophilum* has 5′ to 3′ exonuclease activity, but it is not associated with GINS, whereas RecJ2 has 3′ to 5′ exonuclease activity that is clearly stimulated by GINS. RecJ1 from *Methanocaldococcus jannaschii* has 5′ to 3′ exonuclease activity, and the *recJ1* gene can complement the UV sensitivity of *E. coli recJ* mutant cells^37^. It is not known whether *M. jannaschii* RecJ1 interacts with GINS. RecJ2 from this organism has 3′ to 5′ exonuclease activity, but it does not interact with GINS. The RecJ-like protein from *P. furiosus* (PfRecJ) reportedly showed other different properties. PfRecJ interacts with GINS, and its 5′ to 3′ exonuclease is stimulated by GINS. However, it also has 3′ to 5′ exonuclease activity for RNA, which is not stimulated by GINS. The authors of this report proposed that PfRecJ functions for the proof-reading of the primers generated by primase mistakes^38^. Considering these results, it is difficult at present to propose a unified vision of the properties of the GAN family proteins in Archaea.

Studies of RecJ family proteins are becoming more popular. We have presented the properties of the euryarchaeal-specific HAN protein. It is interesting that two RecJ family proteins, GAN and HAN, may function in progression and repair, respectively, for the replication fork in *T. kodakarensis*. Further studies are necessary to understand the functions of the variously evolved RecJ family proteins.

## Methods

### Site-specific mutagenesis

To construct the pET-21a(+) (Novagen)-based expression plasmid for HAN-D366A and the plasmid to replace the native *han* gene with *han*-*D364A/D366A*, PCR-mediated mutagenesis was performed with a QuikChange^TM^ site-directed mutagenesis kit (Agilent Technologies). The templates were pET-HAN [the gene encoding TkoHAN inserted into the *Nde*I-*Not*I sites of the expression plasmid pET-21a(+)]^29^ and pUD3-HAN (described below). The designed mutations were confirmed by nucleotide sequencing. The primers used for each mutagenesis are listed in Supplementary Table S1.

### Recombinant protein purification

The recombinant HAN (TK_RS00760) and Hef (TK_RS05025) (full-length and His-tagged IDR proteins) were prepared according to the procedures described in our previous reports^29,32^. HAN-D366A was prepared in the same manner as the wild-type protein.

### Nuclease assay

[γ-^32^P] ATP was purchased from Perkin Elmer. The sequences of the unlabeled and fluorescently labeled oligonucleotides are listed in Supplementary Table S2. Some oligonucleotides were labeled at the 5′ termini with [γ-^32^P] ATP by T4 polynucleotide kinase (New England Biolabs). Oligonucleotides used as the DNA substrates were obtained from Hokkaido System Science and Sigma-Aldrich. The combinations of oligonucleotides used as the substrates are shown in Supplementary Table S3. The oligonucleotides were annealed in 20 mM Bis–Tris, pH 7.0, and 50 mM NaCl. The nuclease reaction of HAN was performed in mixtures (20 µl), containing 25 mM Bis–Tris, pH 7.0, 125 µg/ml BSA, 5 mM DTT, 1 mM MnCl_2_, and ^32^P- or FITC-labeled DNA substrates. The nuclease reactions of Hef and HAN-Hef were performed in mixtures (20 µl), containing 25 mM Bis–Tris, pH 7.0, 50 mM KCl, 5 mM DTT, 100 mM MgCl_2_, 0.1 mM MnCl_2_, and Cy5- or FITC-labeled DNA substrates. The reaction mixture was pre-incubated for 3 min without metal ions. The reaction was started by adding metal ions and terminated by adding 5 µl of stop solution A (12.5% Ficoll, 100 mM EDTA, 0.05% bromophenol blue, and 0.05% xylene cyanol) for native polyacrylamide gel electrophoresis (PAGE), or adding an equal volume of stop solution B (98% formamide, 10 mM EDTA, 0.01% bromophenol blue, and 0.01% xylene cyanol) for denaturing PAGE, and immediately transferred onto ice. The gel images were visualized by using a Typhoon Trio+ (GE Healthcare) image analyzer. Further modifications of the reaction and PAGE conditions are described in their respective sections.

### Surface plasmon resonance (SPR) analysis

The Biacore J system (GE Healthcare) was used to examine the physical interactions of HAN–GINS, HAN–Hef (IDR), GAN–GINS, and GAN–Hef (IDR). Either HAN or GAN was fixed on a CM5 sensor Chip (GE Healthcare), according to the manufacturer’s recommendations. The solutions containing purified 2 µM GINS (as the tetramer) and 1 µM Hef-IDR proteins, in running buffer (10 mM HEPES–NaOH, pH 7.4, 150 mM NaCl, 3 mM EDTA, and 0.2% Tween 20), were applied for 120 s to the protein-immobilized chips, at a flow rate of 30 μl/min at 25 °C. The bound analytes were removed by regeneration buffer (10 mM HEPES–NaOH, pH 7.5, 1 M NaCl, and 0.2% Tween 20) at the end of each cycle.

### Strains and culture conditions

*T. kodakarensis* KUW1 (Δ*pyrF*, Δ*trpE*)^48^ was used as the parental host strain for mutant construction. *T. kodakarensis* cells were cultivated under anaerobic conditions at 85 °C or 93 °C, in either nutrient-rich medium (ASW-YT or MA-YT) or synthetic medium (ASW-AA), in basically the same manner as previously described^49–51^. ASW-YT medium was composed of 0.8 × artificial seawater, 5.0 g/l yeast extract (Difco), and 5.0 g/l tryptone (Difco), supplemented with sodium pyruvate (5.0 g/l) or elemental sulfur (5.0 g/l) (ASW-YT-Pr or ASW-YT-S^0^, respectively). For large-scale cultivation, 30.2 g/l artificial sea salts (Marine Art SF; Tomita Pharmaceutical, Naruto, Japan) were used instead of ASW (MA-YT-Pr). The minimal ASW-AA medium, which supported the growth of strain KUW1, and the solid medium were prepared as previously described^49,50^. To select transformants with 5-FOA resistance, 10 g/l 5-fluoroorotic acid (5-FOA) and 60 mM NaOH were added to the solid medium.

### Immunoprecipitation assay

*T. kodakarensis* KOD1 cells were cultured in 2 liters of MA-YT-Pr medium at 85 °C, and harvested at the late exponential phase by centrifugation for 10 min at 5,000 × *g*. The cells (9.1 × 10^11^ cells) were resuspended in 45.5 ml of 50 mM Bis–Tris buffer, pH 7.0, to a final concentration of 2 × 10^10^ cells/ml, and were disrupted by sonication. The cell extracts were obtained by centrifugation for 10 min at 23,000 × *g*. Polyclonal anti-HAN and anti-Hef antisera were raised independently, by immunizing rabbits with the purified recombinant proteins as antigens. A 20 µl portion of rProtein A Sepharose FF (GE Healthcare) was washed three times with phosphate buffered saline-Tween 20 (PBS-T: 10 mM sodium phosphate, pH 7.5, 150 mM NaCl, 0.1% Tween 20), mixed with 500 µl PBS-T containing 100 µl of each antiserum, and incubated on a rotating wheel at room temperature for 1 h. Each mixture was washed three times with 500 µl of 0.2 M triethanolamine, pH 8.0. Each antibody was cross-linked to the rProtein A Sepharose with 10 mM dimethyl sulfide (Thermo Fisher Scientific), according to the manufacturer’s protocol. After equilibration of the antibody-conjugated rProtein A Sepharose with 50 mM Bis–Tris, pH 7.0, a 7.5 ml aliquot of the cell extract was added. The mixture was incubated on a rotating wheel at room temperature for 1 h. The precipitates were washed three times with 50 mM Bis–Tris, pH 7.0, and the immunoprecipitated proteins were eluted with 75 µl of gel-loading solution, containing 50 mM Tris–HCl, pH 6.8, 10% glycerol, 5% β-mercaptoethanol, 0.2% bromophenol blue, and 2% SDS. The eluted proteins were separated by SDS-PAGE and analyzed by western blotting.

### Western blot analysis

The proteins on the gel were electroblotted onto a polyvinylidene difluoride (PVDF) membrane (Bio-Rad) using a Trans-Blot Turbo Transfer System (Bio-Rad), and reacted with the anti-HAN and anti-Hef antisera, prepared with each recombinant protein. Anti-Rabbit IgG HRP (Rabbit TrueBlot, Rockland Immunochemicals, Inc.) was used as the secondary antibody. The proteins were visualized by an enhanced chemiluminescence system (Millipore), and images were obtained and quantified with an LAS-3000 image analyzer (Fujifilm).

### Construction of the *T. kodakarensis* Δ*han*, Δ*gan*Δ*han*, Δ*han::han-D364A/D366A*, and Δ*gan::han-D364A/D366A strains*

The *gan* gene, along with 1,000 bp of its 5′- and 3′-flanking regions, was amplified from *T. kodakarensis* genomic DNA, using the primer sets fhan-F/R (Supplementary Table S4). The PCR fragment was inserted into pUD3^52^ after digestion with *Bam*HI and *Eco*RI, and the resultant plasmid was designated as pUD3-HAN. An inverse PCR to remove the *gan-*encoding region was performed, using the dhan-F/R primer set, and the amplified fragment was self-ligated (pUD3-ΔHAN). The sequences of the 5′- and 3′-flanking regions were confirmed. The transformation was performed as described previously^52^. The Δ*han*Δ*gan* strains were obtained by transforming the Δ*han* cells with pUD3-ΔGAN^25^. The genotypes of the isolated transformants were analyzed by PCR, using han-F/R and fhan-F/R, and the *gan* gene locus of the candidate strains was sequenced to confirm the gene disruption. The plasmid bearing the gene encoding HAN-D364A/D366A (pUD3-HAN-D364A/D366A) was prepared by site-specific mutagenesis. The Δ*han* and Δ*gan* strains were transformed with pUD3-HAN-D364A/D366A, and the strains harboring the nuclease-deficient *han* gene were isolated in the same manner, and designated as Δ*han::han-D364A/D366A* and Δ*gan::han-D364A/D366A*, respectively.

### Growth measurements

The growth characteristics of the *T. kodakarensis* strains KUW1, Δ*gan*, Δ*han*, Δ*gan*Δ*han*, Δ*han::han*-*D364A/D366A*, and Δ*gan::han*-*D364A/D366A* were measured, as follows. Each strain was precultured in ASW-YT-Pr medium at 85 °C (except Δ*gan*Δ*han*) or 70 °C (Δ*gan*Δ*han*) for 6–12 h, until the culture attained an OD_660_ of 0.2–0.3. After preculturing, the cells were inoculated into 15 ml of ASW-YT-Pr to produce an OD_660_ of 0.01, and cultured at 85 and 70 °C in glass test tubes. Cell densities were measured at appropriate intervals at 660 nm (OD_660_), with a Novaspec II spectrophotometer (Pharmacia Biotech). Each strain was analyzed in duplicate, and the measurements were performed three times independently.

### Drop dilution assay

The *T. kodakarensis* KUW1, Δ*han*, and Δ*hef* strains were cultivated anaerobically at 85 °C for 12 h, in ASW-YT-Pr medium. The cultures (5 × 10^8^ cells/ml) were serially diluted ten-fold with 0.8 × ASW. The diluted cultures were spotted on ASW-YT plates, and some of them were irradiated with an acrylic-filtered UV lamp (Kenis) (wavelength 254 nm, 5, 8, and 11 J/m^2^, calculated by an ATV-3W UV counter, ATTO). For exposure to MMC, the cells in 1 ml of culture were collected by centrifugation and resuspended in 100 µl of 0.8 × ASW, and mitomycin C (MMC) (100 µg/ml) was added. The cells were incubated at room temperature for 200 min, and then serial dilutions were spotted on ASW-YT plates. The plates were incubated anaerobically at 85 °C or 93 °C for 24 h, and the living cells were counted and visualized by staining with Coomassie Brilliant Blue (CBB), after transfer to a PVDF membrane, as described previously^53^. Experiments were performed in duplicate each time, and repeated three times independently.

### Data availability

No datasets were generated or analyzed during the current study.

## Electronic supplementary material

Supplementary information
