# Supplementary material for: Possible function of the second RecJ-like protein in stalled replication fork repair by interacting with Hef
Source: Sci Rep. 2017 Dec 5;7:16949. doi: 10.1038/s41598-017-17306-0 (PMC5717133; doi:10.1038/s41598-017-17306-0)
Supplement: Supplementary file 1 — Supplementary information [file 41598_2017_17306_MOESM1_ESM.pdf]

Supplementary Information

Possible function of the second RecJ-like protein in stalled replication fork repair by interacting with Hef

Mariko Nagata[1], Sonoko Ishino[1], Takeshi Yamagami[1], Jan-Robert Simons[2], Tamotsu Kanai[2], Haruyuki Atomi[2], Yoshizumi Ishino[1,*]

[1] Department of Bioscience and Biotechnology, Graduate School of Bioresource and Bioenvironmental Sciences, Kyushu University, Fukuoka, Japan
[2] Department of Synthetic Chemistry and Biological Chemistry, Graduate School of Engineering, Kyoto University, Kyoto, Japan

Supplementary Table S1. Sequences of primers used for cloning and mutagenesis.

primer name	sequence (5′–3′)
HAN_D366A-F	dAGGCATCACGCCGATGCCGcTGGCTATACATCCGGCCTG
HAN_D366A-R	dCAGGCCGGATGTATAGCCAgCGGCATCGGCGTGATGCCT
HAN_D364A/D366A-F	dGGCATCACGCCGcTGCCGcTGGCTATACATC
HAN_D364A/D366A-R	dGATGTATAGCCAgCGGCAgCGGCGTGATGCC

The substitutions are indicated by lowercase letters.

Supplementary Table S2. Sequences of oligonucleotides used in the nuclease assay.

number	name	label	sequence (5′–3′)	length (nt)
1	dA30	none	dCGAACTGCCTGGAATCCTGACGAACTGTAG	30
2	dA30ssss14	none	dCGAACTGCCTggaaTCCTGACGAACTGTAG	30
3	d49N	none	dAGCTACCATGCCTGCACGAATTCGTATCAGCGTAATCATGGTCATAGCT	49
4	d49R	none	dAGCTATGACCATGATTACGCTGATACGAATTCGTGCAGGCATGGTAGCT	49
5	d30	none	dAGCTACCATGCCTGCACGAATTCGTATCAG	27
6	d27	none	dATACGAATTCGTGCAGGCATGGTAGCT	30
7	FITCdA30	5′ FITC	dCGAACTGCCTGGAATCCTGACGAACTGTAG	30
8	dA30FITC	3′ FITC	dCGAACTGCCTGGAATCCTGACGAACTGTAG	30
9	FITCrA30	5′ FITC	CGAACUGCCUGGAAUCCUGACGAACUGUAG	30
10	rA30FITC	3′ FITC	CGAACUGCCUGGAAUCCUGACGAACUGUAG	30
11	FITCrA30ssss14	5′ FITC	CGAACUGCCUggaaUCCUGACGAACUGUAG	30
12	HJ-1-34	none	dGACCGAGCACGCGAGATGTCAACGATCGAATTGC	34
13	HJ-2-59	none	dGCAATTCGATCGTTGACATCTCGCGTGCTCGGTCAATCGGCAGATGCGGAGTGAAGTTC	59
14	HJ-3-54	none	dTCACTCCGCATCTGCCGATTCTGGCTGTGGCGTGTTTCTGGTGGTTCCTAGGTC	54
15	HJ-3-54-FITC	3′ FITC	dTCACTCCGCATCTGCCGATTCTGGCTGTGGCGTGTTTCTGGTGGTTCCTAGGTC	54
16	HJ-4	none	dGACCTAGGAACCACCAGAAACACGCCACAGCCAGGAAGCCGATTGCGAGGCCGTCCTACCATCCTGCAGG	70
17	HJ-4-59	none	dGACCTAGGAACCACCAGAAACACGCCACAGCCAGGAAGCCGATTGCGAGGCCGTCCTAC	59
18	Cy5-HJ-4-59	5′ Cy5	dGACCTAGGAACCACCAGAAACACGCCACAGCCAGGAAGCCGATTGCGAGGCCGTCCTAC	59
19	HJ-4-34	none	dGACCTAGGAACCACCAGAAACACGCCACAGCCAG	34

Small characters represent modifications to phosphorothioate bond at the 5′ side to prevent the 5′ digestion of each phosphodiester backbone.

Supplementary Table S3. Combinations of the oligonucleotides to prepare the substrates.

structure	label	combination
blunt end	^{32}P	3+4
19 nt-5′-overhang	^{32}P	3+5
22 nt-3′-overhang	^{32}P	3+6
sub1	Cy5+FITC	15+18
	Cy5	14+18
	FITC	15+17
sub2	Cy5+FITC	13+15+18
	Cy5	13+14+18
	FITC	13+15+17
sub3	Cy5+FITC	12+13+15+18
	Cy5	12+13+14+18
	FITC	12+13+15+17
sub4	FITC	12+13+15+16
sub5	FITC	12+13+15+19

The numbers on the combination column correspond to those in Supplementary Table S2.

Supplementary Table S4. Primers used for amplification of the *gan* and *han* loci.

name	sequence (5′–3′)
gan-F	dCGCGCATATGGATAAGGAGGCTTTTTTGGAGCGCG
gan-R	dGGGGCGGCCGCTCAACCCTCGCCTTCACTTCCACCG
fgan-F	dAAAGGATCCGAGGTAGCAGAGTTGTATCCTGCGGAG
fgan-R	dAAAGAATTCCGGCTTGAATGTGCTTTGTGGCAGATCC
dgan-F	dGTGGAAGTGAAGGCGAGGGTTG
dgan-R	dGCGGCATCACCGGAAGAAG
han-F	dGGGCATATGGTTGTGAAAGACTGTCCCG
han-R	dGGGGCGGCCGCTTAGGAAATCTTCTTCAGCTTCACG
fhan-F	dAAAGAATTCGCCACCAGCACGTCCTTCCCATTG
fhan-R	dAAAGAATTCGAGTTCTCGTACTGGGGATACCTCTGGC
dhan-F	dTGTCTTTTTCTTTTCCCTGGTTTTGTTCC
dhan-R	dCTTCTCTCACCGGCCTAAGTTTGAAG

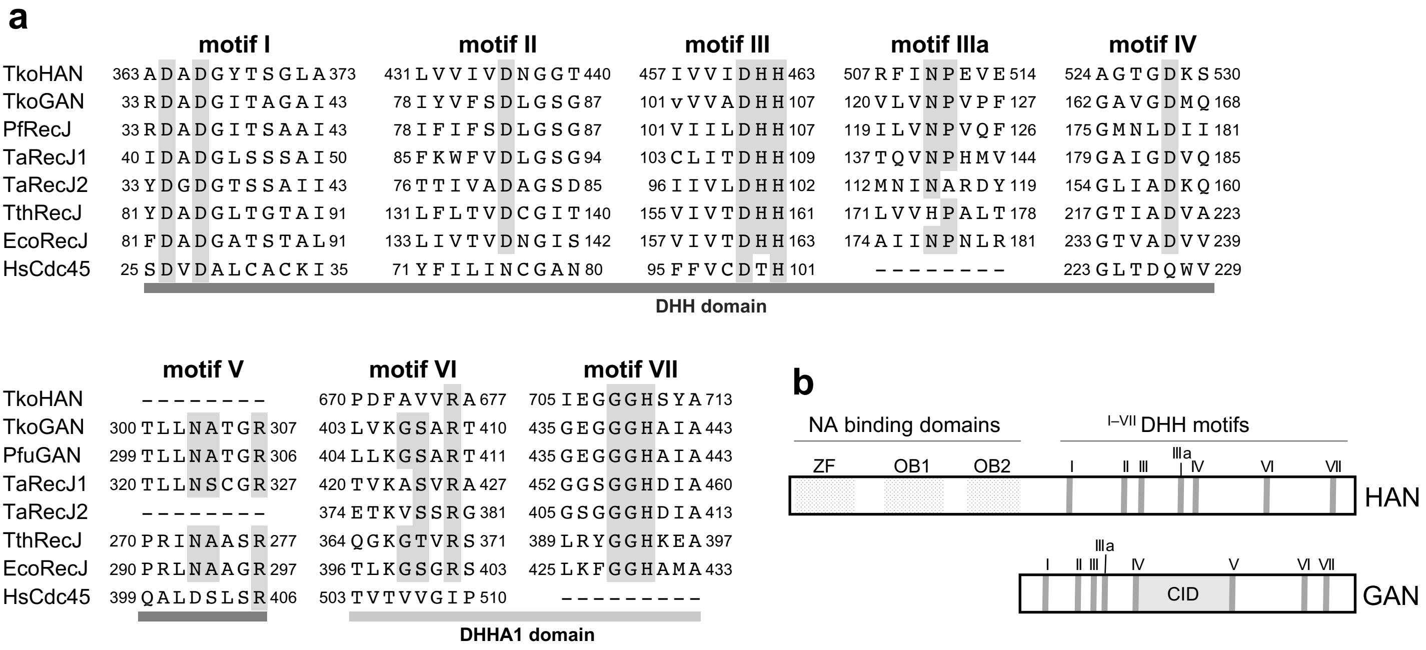

Supplementary Figure S1. Sequence alignment of DHH motifs and domain organizations in HAN and GAN. (**a**) Multiple alignments of the seven DHH motifs in TkoHAN (WP_011249110), TkoGAN (BAD85441.1), and the reported archaeal RecJ-like nucleases from *P. furiosus* (PfRecJ, AAL82179) and *T. acidophilum* (TaRecJ1, WP_010900953; TaRecJ2, WP_010901541), bacterial RecJs from *T. thermophilus* (TthRecJ, BAB61865) and *E. coli* (EcoRecJ, AAA62789), and eukaryotic Cdc45 from *Homo sapiens* (Hs, AAC67521.1). Sequences were aligned by using CLUSTALW, and were further adjusted manually. (**b**) Schematic representations of the GAN and HAN proteins, showing each domain. The lengths of the bars, and the positions of the motifs and domains correspond to those in *T. kodakarensis* (TkoHAN and TkoGAN). The positions of motifs I–VII are indicated by bars. HAN has three NA binding domains at its N-terminus and active DHH motifs at its C-terminus.

Zinc finger I

CxxCxGxG CxxCxGxG

```
|WP_011249110.1| Thermococcus kodakarensis                MVVKDCPECHGTGKVKVGEKECPVCEGWGYVPADFKIGDKLKGYRN--LDHLGVEDEV-D 57
|WP_011011512.1|Pyrococcus furiosus                       MAVKDCPECGGSGKVKVGEKECSVCNGWGYVPADFKLTDHLKGYRN--LENFGVDEEV-D 57
|WP_048060894.1|Methanothermobacter thermautotrophicus    -MIQSCNECKGKGYRVKSYKICSACHGTGFR-STEDIKDHFKGVSNSARQRFDLEDS--H 56
|Q58598.1|Methanocaldococcus jannaschii                   -MIVKCPICDGTGKKVVKYKTCPVCEGTGFI-DEFSPKQHMKRVSK--RATYDLDY---G 53
|WP_010878834.1|Archaeoglobus fulgidus                    ---MICNACGGKGYIEI-EKECEICGGTGKA-KSFDPKITAE------LSDEQIKMF--M 47
|WP_048064996.1|Methanosarcina acetivorans                -MSKECPDCHGRGYEVISTEVCPLCKGKGKS-KSVDFMK---------ISEKDIDSFLKN 49
|WP_014406706.1|Methanocella conradii                     -MSLECPSCSGKGLVIKGEKPCENCNGTGKV-KSVNLIG---------MTEKDLKSLL-S 48
|AAM01374.1|Methanopyrus kandleri                         MPREKCPKCDGKGKIPVGETECPRCGGTGFV-GDVDISEHFKGAAQHAVEGYDLASS--R 57
|WP_004043924.1|Haloferax volcanii                        ------------------------------------------------------------
```

Zinc finger II

CxxCxGxG CxxCxGxG

```
|WP_011249110.1| Thermococcus kodakarensis                EIPCPECHGKGTVPVYDTCPTCGGTGKVLACDICGKIKGPWEPGMETTWVCPDCLRKYKV 117
|WP_011011512.1|Pyrococcus furiosus                       EIPCPECHGKGTVPVYDTCPTCGGTGKVLACDICGKVKGPWEPGMETTWVCPECERKFKV 117
|WP_048060894.1|Methanothermobacter thermautotrophicus    EVPCEVCRGKGEVEVRETCPTCGGKGEVNICPSCGRMM------KGRDEYCPECQKKEK- 109
|Q58598.1|Methanocaldococcus jannaschii                   EIPCPKCKGTGKVPVYAKCDFCGGSGKVVKCDRCGAIIGKY-PDFKDRTLCDKCLKEEEE 112
|WP_010878834.1|Archaeoglobus fulgidus                    SGVCGVCRGTGKVKIMDVCRECNGTGKAGRCKICGEKV------VGNHDLCSRCRRQPH- 100
|WP_048064996.1|Methanosarcina acetivorans                GAVCEKCKGKGSVEVTRPCEACEGLGKIYTCKICGVRIHD--PQEAEEEICSSCARSQH- 106
|WP_014406706.1|Methanocella conradii                     GGFCPKCKGSGKILVKEKCPDCGGTGKVPVCEICGKAVAP------GQSLCESCINVRP- 101
|AAM01374.1|Methanopyrus kandleri                         DVPCPKCQGKGVITVYEECDRCGGTGYIVKCRECGKELD---PDVEED-LCEECRKKIKQ 113
|WP_004043924.1|Haloferax volcanii                        ----------------------------------------------MSDEHAGDSGDDSG 14
```

OB-fold I

```
|WP_011249110.1| Thermococcus kodakarensis                --------VYILDKTCDYEDVEVGSLYKGTIDRVERFGVFVKLNPHVTGLIKRKDLLGGR 169
|WP_011011512.1|Pyrococcus furiosus                       --------VYVLDNACDYEDVEIGSYYKGVIDRVERFGVFVKLNKHVIGLIKKKDLLGKR 169
|WP_048060894.1|Methanothermobacter thermautotrophicus    --------VYILHPACTMDDLEVGSIYRGKITRVEKYGVFVSLNSHVWGLM--RGLFP-- 157
|Q58598.1|Methanocaldococcus jannaschii                   RKKGL-RNVYVFDELATFYDVEPGKFYKGVVTRIEKYGAFINLNEQVRGLLRPRDMISLR 171
|WP_010878834.1|Archaeoglobus fulgidus                    --------AYRLRNSCGIEDVRINRVYVGTVSAVTDIGVFVNLNKRLRGLIHRRNLGNNR 152
|WP_048064996.1|Methanosarcina acetivorans                --------VYALDESCDLKDVEAGKLYHGIVSSIASFGVFVDLNPHVRGLMHSSNVGV-- 156
|WP_014406706.1|Methanocella conradii                     --------VYKLSAACDVSDLDAGKTYLGRVANLADFGVFVSLNDQTKGLIHSSNVNR-- 151
|AAM01374.1|Methanopyrus kandleri                         IRKEKLPKVKVLSPACGYEDVEEGELYKGKVSRVEKYGVFIELNDRTLGLLHRRDMGDKE 173
|WP_004043924.1|Haloferax volcanii                        PNSDARPIVYDLAPNCTADDVETDAYYHAVVNGVVEYGIFVDVSDSVSGLIHESNLSA-- 72
```

```
|WP_011249110.1| Thermococcus kodakarensis                --EYKPGDEIVVQVLDVRPDKREVDL----IESALKH-YKTVVVRKELPVTPIAELSK-- 220
|WP_011011512.1|Pyrococcus furiosus                       --EYKPGDEIIVQVLDVRPDKREIDF----LEAPLTK-YREVHVKKELPVTPIEELKE-- 220
|WP_048060894.1|Methanothermobacter thermautotrophicus    --DYKVGDELFVRVSQVKPYKGEVDM----IPASIKGPYEVIKLKKDLPRTRIADIDT-- 209
|Q58598.1|Methanocaldococcus jannaschii                   LENLNVGDEIIVQAIDVRPEKREIDF----KYIPLTT-YDLVKYEKEVPLSQIKDISQNL 226
|WP_010878834.1|Archaeoglobus fulgidus                    ---FSEDEEILVQVSGIGLS-GEIDL----KPVKMDG-YKVVEISKEVGRVEIAELEN-- 201
|WP_048064996.1|Methanosarcina acetivorans                --PPEVGEALIVLVKSIKAG-GKLDL----IPKTLTK-YETIELEKELPLKDSSEIDT-- 206
|WP_014406706.1|Methanocella conradii                     --PYVPGEEVMVKVNTIKPN-GNFDL----IPQRVSE-FKLIEVEKSLPRRKSSEVGK-- 201
|AAM01374.1|Methanopyrus kandleri                         PQDFSIGDEVVVKVTDVRPEDGEIDFTIEGIDPRPDR-YREEVVEKELKRVLVHDIDE-- 230
|WP_004043924.1|Haloferax volcanii                        --DYEVGDRLVVRLESVRDN-GDIAF----AEDTPDD-YRTLTVDHEPTITPISSLSV-- 122
```

OB-fold II

```
|WP_011249110.1| Thermococcus kodakarensis                -DMAGKTVRIQGRITQVQVTGGPTVFTITDGTGITWVAAFEAPGVRAYPNINVGDIVEVI 279
|WP_011011512.1|Pyrococcus furiosus                       -ELAGRTVKIRGKVTQVQVTGGPTVFTLTDGTGITWAAAFEAPGVRAYPNINVGDIVEII 279
|WP_048060894.1|Methanothermobacter thermautotrophicus    -KSLGKTVRIVGEVIQIQQTSGPTIFTVSDETGTTWAAAFDEPGIRVYPHIQIGHIVEVI 268
|Q58598.1|Methanocaldococcus jannaschii                   VEMRDQVVHIRGEVVQIVQTPGPTVFTITDGTDFAWVAALEIAGLRAHPDVKVGDIVDVI 286
|WP_010878834.1|Archaeoglobus fulgidus                    -YI-GKMVEVRGLVTHIKVTGGPTIFTLLDGRASVQAAAFEG-GERAYPEVRVDDVVRVI 258
|WP_048064996.1|Methanosarcina acetivorans                -SMKGRLIRIEGEVIQVKQTSGPTIFTISDEGGFIPCAAFESAGKRSYPHIDVGMIVSIT 265
|WP_014406706.1|Methanocella conradii                     -FV-GKLVSISGEVVQIKQTSGPTIFTIADEDGVVSCAAFTEAGMRAYPEINLEEIVRVT 259
|AAM01374.1|Methanopyrus kandleri                         -SKIGETVLIKGKIIHVQQTPGPTVFTLRDESGSIWMAAFEGPGIRAYPDIEAGDYVRVI 289
|WP_004043924.1|Haloferax volcanii                        ----GESVTVEGVVTQIKQTGGPTVFRIADDSGIVAAAAFEEAGVRAFPGVDLDDVVRMA 178
```

```
|WP_011249110.1| Thermococcus kodakarensis                GKVAFHSGEIQIEASDMARLWGPDAARVKQQIEAELDRRAQPE-DVGFLVESEVLEKLKP 338
|WP_011011512.1|Pyrococcus furiosus                       GKVSFHAGSIQVEVIDMYRLWGPDAAEVKRKIEEELDRRAQPS-DVGFLVKSEVLEALKP 338
|WP_048060894.1|Methanothermobacter thermautotrophicus    GEVNQHTGKIQIESESIERLIGNEAAEARRLIDEAIDRRAEPE-RKDLLIESETLEKLRP 327
|Q58598.1|Methanocaldococcus jannaschii                   GRVTIRDGRLQIERIKLQKLEGDEAEEIRKKIEEEIDRRAEPAKDIPFLVKSEVLERLRP 346
|WP_010878834.1|Archaeoglobus fulgidus                    GIVKRRENKLQIEILEMEKLLGEEAYEVRKRVEAEIERACEPD-FRGFLIESEVLEALKE 326
|WP_048064996.1|Methanosarcina acetivorans                GEVTPRDEQVQIEVMSMKLLTGEKEAAVKFRVEKVIEEKAAPA-DIPFLVESAIMERLKP 315
|WP_014406706.1|Methanocella conradii                     GEIEMHNGSLQMEVLEMKKLSVADAAPIREVIERSIDARAAPA-HVEFLIKSEALERLRP 318
|AAM01374.1|Methanopyrus kandleri                         GEVTTHDGQLQVEILDMEKLVGTEKVEIKRAIDEALDREAEPPEDLKPMVDSEIIERLWP 349
|WP_004043924.1|Haloferax volcanii                        GTVEDHEGTRQLEVDGLTVLDDEAAADARQRIDAALDERAEPE-PVEPLVEWSAFEKLRD 237
```

Supplementary Figure S2a.

motif I

```
|WP_011249110.1| Thermococcus kodakarensis                 KIMKAAFMIRRAILEGRPILLRHHADADGYTSGLALEYAIVPLIEKISPDPQARWKLFKR 398
|WP_011011512.1|Pyrococcus furiosus                        KIMKAAFMIRRAIFEGRPIILRHHADTDGYTAGVALETAIIPLIEKVAPDPEARWHLFKR 398
|WP_048060894.1|Methanothermobacter thermautotrophicus     KLVEAAKAIRRAVYDGRSILVRHHADADGICAGVAIEKAVLPLIRDLNPSTDAEWHYFKR 387
|Q58598.1|Methanocaldococcus jannaschii                    KMADVAKRIRKAVLDGRPIIIRHHADTDGYCGGIALEKAILPIIDKFAIDVDAIWHFFKR 406
|WP_010878834.1|Archaeoglobus fulgidus                     DMLKVAKELKKAIYESRPVIIRHHWDADGTCGGVALEKALTDLVERVHSDSEAKYYLVKR 377
|WP_048064996.1|Methanosarcina acetivorans                 RMLHVAKEIKKAIFHSTPIILRHHADADGITSAIAIERAILPLITEI-GGADAEYYFYKR 383
|WP_014406706.1|Methanocella conradii                      QMEKVARRIRRAIFKSEPIIVRHHADADGICAGVAIEKACLPLI-KAQGDLDAEYHFFSR 377
|AAM01374.1|Methanopyrus kandleri                          RMREVAKEIKRAVLEGRPVLIRHHADADGISGGVALEEAILPIIRENNPDPEAEYHFYKR 409
|WP_004043924.1|Haloferax volcanii                         DLEDVARLLRRTVLEGRPIRVRHHADGDGMCASVPVQLALERLITEVHDDPDAPRHLFKR 297
```

motif II

```
|WP_011249110.1| Thermococcus kodakarensis                 RPSRAPFYELEDVLKDIIFMVEDHEKFGDPLPLVVIVDNGGTSEDIPAYKRIRAYGVPIV 458
|WP_011011512.1|Pyrococcus furiosus                        RPSRAPFYELEDVLKDIIFMMEDHMRFGDELPLVVIVDNGGTTEDIPAYKRLKAYGVKIV 458
|WP_048060894.1|Methanothermobacter thermautotrophicus     APSKAPFYELEDVVKDLSYALEDLERHGQKLPLLVLLDNGSTEEDILALMKAKIYDIEIV 447
|Q58598.1|Methanocaldococcus jannaschii                    RPSKAPFYELEDVTKDLVFSIEDALKFGQKLPLIVLIDNGSTDEDIPAISKAKAYGIEVI 466
|WP_010878834.1|Archaeoglobus fulgidus                     RVSRAPFYELEDVVRDLDESLEDAERHGDKIPLVVLVDNGSGLEDVPAIRQFLLFGADVI 437
|WP_048064996.1|Methanosarcina acetivorans                 APSKAPFYELADVTRDISFALEDYARHGQKMPLVILVDNGSTEEDVPSMRQAKVYGINML 443
|WP_014406706.1|Methanocella conradii                      SPSKAPFYELEDVNKDIAMALEDRARFGQAMPLILLMDNGSTEEDLDAFKYAQVYGIDLM 437
|AAM01374.1|Methanopyrus kandleri                          FPNKAPIYTLEDASRDLNHALEDVHRYGHQVPLLVLLDIGCTEEDVPAIEEMKAYGVDVL 469
|WP_004043924.1|Haloferax volcanii                         LPSKAPFYEMEDVTRDLNFALEGRARHGQRLPFLLMLDNGSTEEDVPAYENLAHYDVPIA 357
```

motif III motif IIIa

```
|WP_011249110.1| Thermococcus kodakarensis                 VIDHHDPREWVSEDKAK------VDDYVDVHVNPHHVKRGYYELTAGMLATEVARFINPE 512
|WP_011011512.1|Pyrococcus furiosus                        VIDHHDPRDWISEDKAK------VDEYVDVHVNPHHVKRGYYELTAGMLATEVARYINPE 512
|WP_048060894.1|Methanothermobacter thermautotrophicus     VVDHHYPGEV-QDGRVE------VDEYVDTHVNPYLVG-GDSQITAGSLSVEIAKMINPE 499
|Q58598.1|Methanocaldococcus jannaschii                    VIDHHFPGEVV-DGKVE------VDDYVDAHVNPYLVG-GDSNLTAGVLGTEIARMINPD 518
|WP_010878834.1|Archaeoglobus fulgidus                     TIDHHFPDEE-------------VDSYLLYHVNPYKVG-GDSNYTSGVLCVEIARMIS-- 481
|WP_048064996.1|Methanosarcina acetivorans                 VVDHHHPDDI-------------VDQYLIGHANPAHVG-GDFGVTAGMLCAEIARMINPG 489
|WP_014406706.1|Methanocella conradii                      VVDHHHPDEV-------------VDQYLQAHVNPYHVG-ADFGITAGMLGTELARMINPD 483
|AAM01374.1|Methanopyrus kandleri                          VIDHHYPGEAVGENPEDGLKEFPIDEHVKVHVNPYAAGGDGKNIPAGVLAVEIARLINPE 529
|WP_004043924.1|Haloferax volcanii                         VVDHHHPDPEA------------VDPLLDAHVNPYLHD-EDYRITTGMMCVELARMIHPD 404
```

motif IV

```
|WP_011249110.1| Thermococcus kodakarensis                 VEDKIKHLPAIAGTGDKSKAPEFYQYLEIAKK-----------AKGLTEEDLKKIAEVID 561
|WP_011011512.1|Pyrococcus furiosus                        VEDRIKHLPAIAGTGDRSKAPEFYQYLEYAKE------------KGLDEEDLKKIAEVID 560
|WP_048060894.1|Methanothermobacter thermautotrophicus     IAERILHLPGIAAVGDHANSPEAEAYIELAAE------------RGYEREDLERIAACID 547
|Q58598.1|Methanocaldococcus jannaschii                    VEDEIKHIPGIAVVGDHAKGEEAEQYVKIALDRLNELSKKYGKGRTYDREYLEKIALCMD 578
|WP_010878834.1|Archaeoglobus fulgidus                     -DLDMKHLAAISVVGDRAEG-EVERYIELSGK---------------SREELADIALAVE 524
|WP_048064996.1|Methanosarcina acetivorans                 ISDTIKHLPAVSAVGDRSEAPEAGRYISLVSD-------------RYTLGELKDMALALD 536
|WP_014406706.1|Methanocella conradii                      IGEKIMHLAAVAAVGDRSEAPEREQYVGLVKD-------------RYSEEDLKNMALALD 530
|AAM01374.1|Methanopyrus kandleri                          VEDRIKHLPAVACLGDHAESPEAEQYLEIAEE------------AGFDRKWLRKIADSVD 577
|WP_004043924.1|Haloferax volcanii                         VTDELRHVPAVAGLSDRSKAEVMDNYIALAED------------EGYDREQLLDVGEALD 452
```

```
|WP_011249110.1| Thermococcus kodakarensis                 HEAYFWKFMDGHGIIDEILLL-TGNLQRHRELINAIYPEVKEKQEKALRASLPHVKSVVL 620
|WP_011011512.1|Pyrococcus furiosus                        HEAFYWKFMDGRGIIEEILLI-TGNLQRHRMLVEGIYPEVKEKQEKVLKAVLPHVKSVVL 619
|WP_048060894.1|Methanothermobacter thermautotrophicus     FEAFYLRFMNGRGIIDTILG--LGSLDKHKKLVDALYREYERKVDTQLRAAIPNLKSTRL 605
|Q58598.1|Methanocaldococcus jannaschii                    FEAFYLRFMDGKGIVDDILATNIKEFGRHEELIDILYEQAMKMVERQMKAVIPALKTEFL 638
|WP_010878834.1|Archaeoglobus fulgidus                     YEGFYLRFRTASQIMHEILG--FGRQDRHVKLVRMLSEYAKEAIEEQVKTAMEGVKVQIL 582
|WP_048064996.1|Methanosarcina acetivorans                 YEQFWLKFSSGKGLIDDILD--LGDHKVHKNLVSLLCEQANTMIKEQLETCLFNVKSQKL 594
|WP_014406706.1|Methanocella conradii                      YEQFWLRYNDGKGIVDDILD--LGNEGRHKALVSLLAGQAARMISDQLEASLPHVKSVEL 588
|AAM01374.1|Methanopyrus kandleri                          FQAFQLRHTPGRHLMNDVLGT-TGDEHRHRRLVENLYKQHKIACERQLEAALKGVK-EYE 635
|WP_004043924.1|Haloferax volcanii                         YAAHWLRYNDGASIVNDVLNVGCDDEERHRELVEFLSTRAERDVDRQLEAAEPHLEHERL 512
```

motif VI

```
|WP_011249110.1| Thermococcus kodakarensis                 PNGVRFNTIDVELFAPKFSYPSPGKLSGLIHDHFKEKYGEDSPILTLAYGPDFAVVRASD 680
|WP_011011512.1|Pyrococcus furiosus                        PNGIRFNTIDVELYAPKFEYPSPGKLSGIIHDHFKEQYGEDSPILTLAYGPDFAVVRASD 679
|WP_048060894.1|Methanothermobacter thermautotrophicus     PNGILFNVLDVEKYSHRFTFPAPGKTCGFVHDYMVQKHGEDTPIITLAYGPDFGVIRATD 665
|Q58598.1|Methanocaldococcus jannaschii                    ENGIILNTLDVEKYAHKFTFPAPGKTTGFAHDYIVQKYGEDKPIITLSYGPDFGVVRATD 698
|WP_010878834.1|Archaeoglobus fulgidus                     PNGIALAALDVENYAKKFTFPPPGKLTGEVHDRLKQKY-E--RIVTIGYGPDFAVIR-SE 638
|WP_048064996.1|Methanosarcina acetivorans                 ANGAIMNVIDVENYAQKFTFPPPGKTSGEMHDVLCKRN-PDKPVVTIGYGPDFAVIR-SK 652
|WP_014406706.1|Methanocella conradii                      PNGAMLHVIDVENYAHKFTFPAPGKTTGEIHDLLCKKY-EGKPTVTLGYGPDFAILR-SR 646
|AAM01374.1|Methanopyrus kandleri                          TDGVKVVTLDVEKHARKFEYPGPGKTCGLVHDLKVEEEGDDAKVVTIAYGPDFAVIRATE 695
|WP_004043924.1|Haloferax volcanii                         DSGANLYRIDLDKWAHRFTYPAPGKTTGKLHDRKVQETGE--PVITIGYGPDFAVLR-SD 569
```

motif VII

```
|WP_011249110.1| Thermococcus kodakarensis                 GM-AAYGFDLNEIIPKLQEKLPSAGIEGGGHSYAGSIKFFEGMRKEVLEEFAKEVVKLKK 739
|WP_011011512.1|Pyrococcus furiosus                        GM-AKYNFDLNKIVKILAEKLPDAGVEGGGHSYAGSIKFFEGKRKEVLEAFAKEVLKLKA 738
|WP_048060894.1|Methanothermobacter thermautotrophicus     AVNEKFGFNLNEIVWELAEEIPEAVIDGGGHECAGSLKYIEGLSKKVLSAFAEKVAALKG 725
|Q58598.1|Methanocaldococcus jannaschii                    AVHEKYNFNLNLIVEQLMEEIPEASLDGGGHECAGSLKFVEGLRDKVIGRFIEIIKNMKP 758
|WP_010878834.1|Archaeoglobus fulgidus                     GV----ELDIPRIVKELREEIV-AGVDGGGHLVVGSIKFVQAKRKEVLARLAAKIGNLK- 692
|WP_048064996.1|Methanosarcina acetivorans                 GV----LMNIPRIVRELHEEMKGAGVSGGGHLVVGSIKFVEGMRTEVLSRLAEKIASTEV 708
|WP_014406706.1|Methanocella conradii                      GV----QMNIPKMVRELREEVRGGGVNGGGHLVVGSIKFVEGMRKEVLEKLTQKIGRAPI 702
|AAM01374.1|Methanopyrus kandleri                          NL----GINLNDIVSELEEEMPEAAVEGGGHETAGSISFVEAHRNKVLKALVEKILRDAT 751
|WP_004043924.1|Haloferax volcanii                         GV----RLDIPRMVAELNEEVVGGGVSGGGHLVVGSIKFVSGMREEVIDSLVEKMAEADI 625
```

```
|WP_011249110.1| Thermococcus kodakarensis                 IS------ 741
|WP_011011512.1|Pyrococcus furiosus                        GE------ 740
|WP_048060894.1|Methanothermobacter thermautotrophicus     -------- 725
|Q58598.1|Methanocaldococcus jannaschii                    KEQ----- 761
|WP_010878834.1|Archaeoglobus fulgidus                     -------- 692
|WP_048064996.1|Methanosarcina acetivorans                 EY------ 710
|WP_014406706.1|Methanocella conradii                      G------- 703
|AAM01374.1|Methanopyrus kandleri                          S------- 752
|WP_004043924.1|Haloferax volcanii                         DEELSTTA 633
```

Supplementary Figure S2a (continuation).

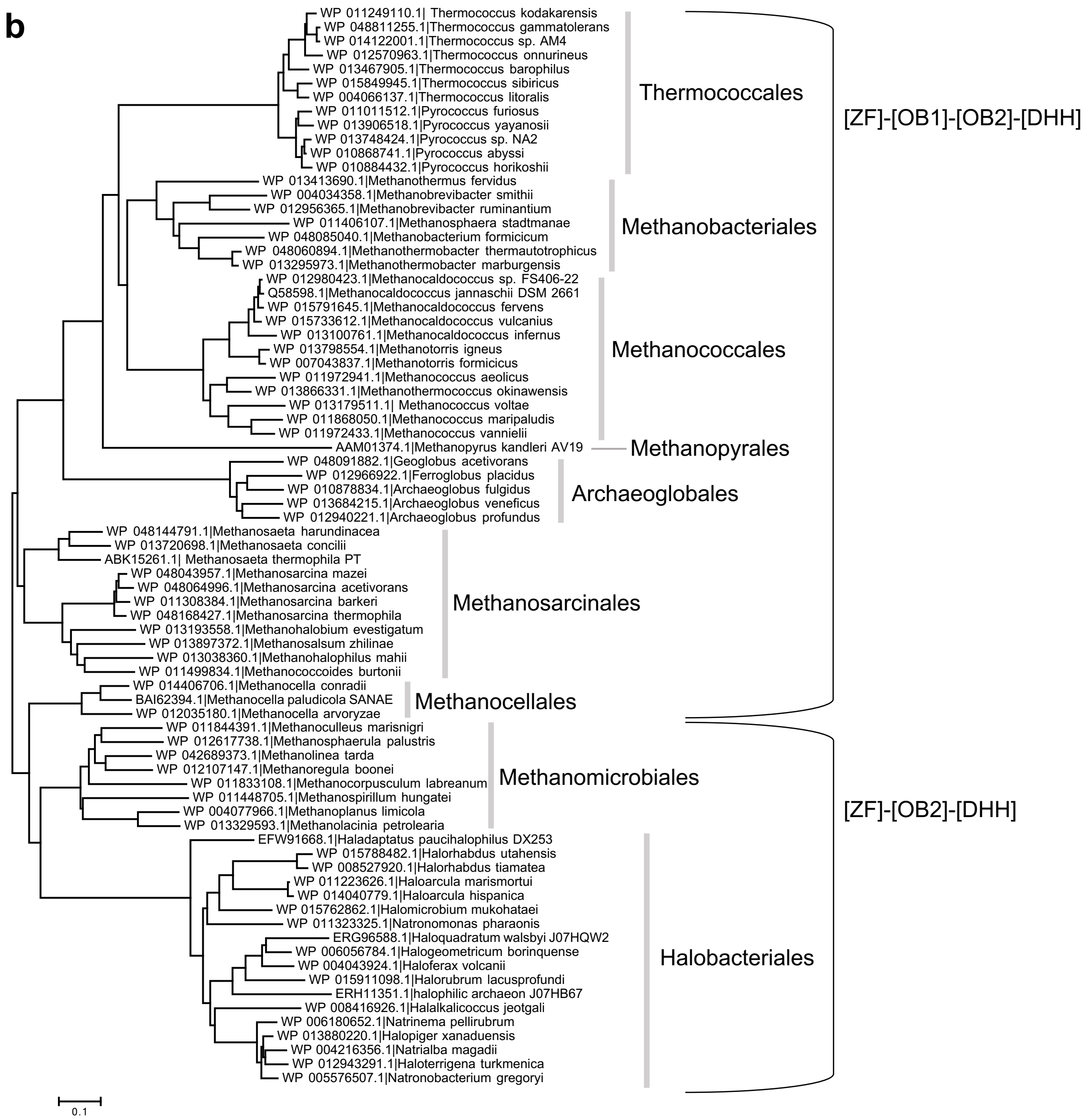

Supplementary Figure S2. HAN homologs in the Euryarchaeota phylum. (**a**) A multiple alignment of the amino acid sequences of the HAN homologs. The amino acid sequences of the HAN homologs were obtained by a BLAST search of the non-redundant protein sequence database at the NCBI site. The amino acid sequence of HAN from *T. kodakarensis* (WP_011249110) was used as the query. Several representative sequences were selected from each taxonomic order[1]. Framed areas are well conserved regions among HAN homologs. Multiple amino acid sequences were aligned using MUSCLE. Predicted secondary structural β-barrels, selected according to that in TkoHAN by Jpred4, are indicated by orange arrows on the top of the sequences. (**b**) An unrooted phylogenetic tree of HAN homologs. To draw the phylogenetic tree, the evolutionary history was inferred using the Neighbor-Joining (NJ) method[2]. The tree is drawn to scale, and the branch lengths have the same units as those of the evolutionary distances used to infer the phylogenetic tree. The evolutionary distances were computed using the JTT matrix-based method[3], and their units are the number of amino acid substitutions per site. The rate variation among sites was modeled with a gamma distribution (shape parameter = 1). The analysis involved 77 amino acid sequences. Evolutionary analyses were conducted with MEGA7[4].

references

1. Petitjean, C., Deschamps, P., Lopez-Garcia, P., Moreira, D. & Brochier-Armanet, C. Extending the conserved phylogenetic core of archaea disentangles the evolution of the third domain of life. *Mol Biol Evol* **32**, 1242-1254, doi:10.1093/molbev/msv015 (2015).

2. Saitou, N. & Nei, M. The neighbor-joining method: a new method for reconstructing phylogenetic trees. *Mol Biol Evol* **4**, 406-425 (1987).

3. Emile Zuckerkandl & Pauling, L. Evolutionary divergence and convergence in proteins. *Evolving Genes and Proteins*, 97–166 (1965). 4. Kumar, S., Stecher, G. & Tamura, K. MEGA7: Molecular Evolutionary Genetics Analysis version 7.0 for bigger datasets. *Mol Biol Evol* **33**, 1870-1874, doi:10.1093/molbev/msw054 (2016).

4. Kumar, S., Stecher, G. & Tamura, K. MEGA7: Molecular Evolutionary Genetics Analysis version 7.0 for bigger datasets. *Mol Biol Evol* **33**, 1870-1874, doi:10.1093/molbev/msw054 (2016).

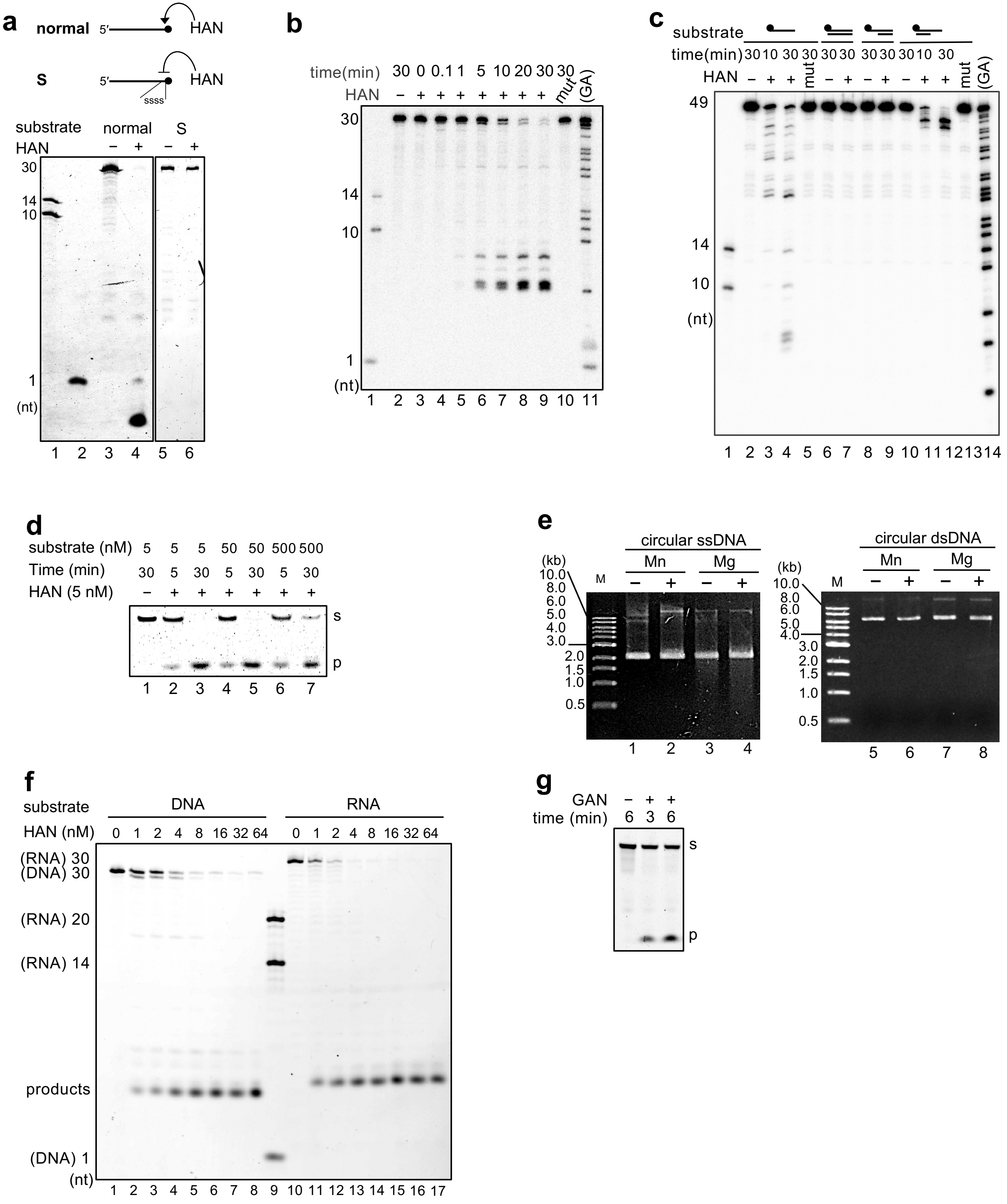
a
normal
5′
HAN
S
5′
HAN
ssss
substrate
normal
S
HAN
30
14
10
1
(nt)
1 2 3 4 5 6
b
time(min)
30 0 0.1 1 5 10 20 30 30
HAN
mut
(GA)
30
14
10
1
(nt)
1 2 3 4 5 6 7 8 9 10 11
c
substrate
time(min)
30 10 30 30 30 30 30 30 30 10 30
HAN
mut
mut
(GA)
49
14
10
(nt)
1 2 3 4 5 6 7 8 9 10 11 12 13 14
d
substrate (nM) 5 5 5 50 50 500 500
Time (min) 30 5 30 5 30 5 30
HAN (5 nM)
s
p
1 2 3 4 5 6 7
e
circular ssDNA
Mn
Mg
(kb)
10.0
8.0
6.0
5.0
4.0
3.0
2.0
1.5
1.0
0.5
M
1 2 3 4
circular dsDNA
Mn
Mg
(kb)
10.0
8.0
6.0
5.0
4.0
3.0
2.0
1.5
1.0
0.5
M
5 6 7 8
f
substrate
DNA
RNA
HAN (nM) 0 1 2 4 8 16 32 64 0 1 2 4 8 16 32 64
(RNA) 30
(DNA) 30
(RNA) 20
(RNA) 14
products
(DNA) 1
(nt)
1 2 3 4 5 6 7 8 9 10 11 12 13 14 15 16 17
g
GAN
time (min)
6 3 6
s
p

Supplementary Figure S3. Exonuclease activity of HAN

(**a**) Exonuclease activity of HAN for 3′-labeled DNA. Illustrations of the substrates with or without phosphorothioate modification and the cleavage site are shown on the top of the panel. The structures of the DNA substrates, with the position of the FITC-label (black circle) at 3′-terminus, are illustrated. HAN (5 nM) was incubated with 3′-FITC-labeled dA30 (5 nM) at 70° C for 30 min. The reaction products were analyzed by 8 M urea-15% PAGE. Although HAN cleaved 3′-FITC-labeled ssDNA without phosphorothioated modification (“normal”, lane 4), 3′-FITC-labeled ssDNA with 4-nt-phosphothioated modification at 3′ terminus (“S”) was not cleaved by HAN (lane 6). Lane 1 shows 5′-FITC-labeled DNA (20, 14 and 1 nt) size markers.

(**b**) Time course experiment of the exonuclease activity of HAN for 5′-^{32}P-labeled ssDNA. HAN (5 nM, WT and D366A mutant) was incubated with 5′-^{32}P-labeled 5 nM ssDNA at 70° C for 0.1, 1, 5,10, 20, and 30 min. The reaction products were analyzed by 8 M urea-15% PAGE.

(**c**) Structure specificity of HAN nuclease. HAN (50 nM, WT and D366A mutant) was incubated with 100 nM 5′-^{32}P-labeled ssDNA (d49N, lanes 2–5) and its annealed products, dsDNA (d49N/d49R , lanes 6 and 7), 5′-overhang (d49N/d30, lanes 8 and 9), and 3′-overhang (d49N/d27, lanes 10–13), at 50° C for 10 and 30 min. The reaction products were analyzed by 8 M urea-15% PAGE. The structures of the DNA substrates, with the position of the ^{32}P-label (black circle), are illustrated on the top. Lanes 1 and 14 show the size markers (10 and 14 nt) and the GA ladder made of the substrate DNA (d49N), respectively.

(**d**) Processive cleavage of HAN nuclease. HAN (5 nM) was incubated with 5′-FITC-labeled dA30 (5 nM) and non-labeled dA30 (0, 50, 500 nM) as a competitor to protect reloading of HAN onto DNA at 70° C for 5, 30 min. The reaction products were analyzed by 8 M urea-15% PAGE. The band assignments are indicated on the side of the panel: s, substrates; p, cleaved products.

(**e**) HAN lacks endonuclease activity. HAN (120 nM) was incubated with 18 ng/μl of pBlueScript II SK(+) ssDNA (lanes 1–4) or 12 ng/μl of M13mp18 dsDNA (lanes 5–8) at 70° C for 30 min. Reaction products were analyzed by 1% agarose gel electrophoresis, followed by SYBR-Gold staining.

(**f**) HAN possesses both DNase and RNase activities. HAN (1–64 nM) and 10 nM 5′-FITC-labeled DNA or RNA substrates were incubated at 80° C for 3 min. The substrates were DNA (FITCdA30, lanes 1-8) and RNA (FITCrA30, lanes 10–17), and the reaction products were analyzed by 8 M urea-15% PAGE. Lanes 2–8 and lanes 11–17 contained 1, 2, 4, 8, 16, 32, and 64 nM HAN, respectively. Lane 9 shows 5′-FITC-labeled RNA (20 nt and 14 nt) and DNA (1 nt) size markers.

(**g**) Confirmation of the DNase activity of GAN used for this study. Corresponding to Figure 1d. GAN (5 nM) was incubated with 3′-FITC-labeled dA30 (1 μM) at 60° C for 3, 6 min. The reaction products were analyzed by 8 M urea-15% PAGE. The band assignments are indicated on the side of the panel: s, substrates; p, cleaved products.

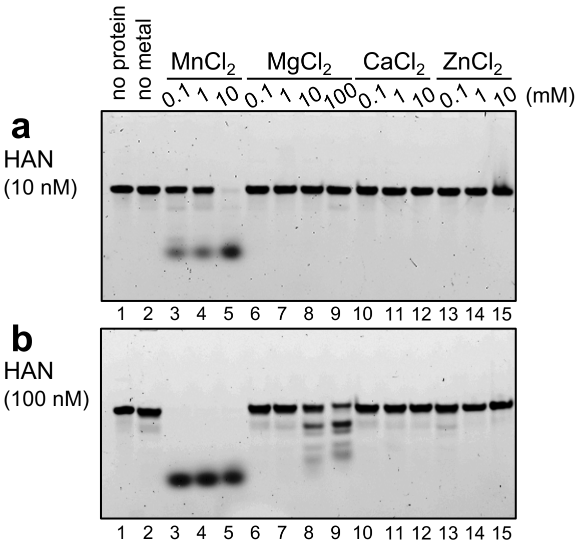

Supplementary Figure S4. The effect of divalent ions on the nuclease activity of HAN. HAN [(**a**), 10 nM; (**b**), 100 nM] was incubated with 10 nM 5′-FITC-labeled DNA (FITCdA30) in 20 μl reaction mixtures containing various metal ions at 80° C for 10 min. The reaction products were analyzed by 8 M urea-15% PAGE.

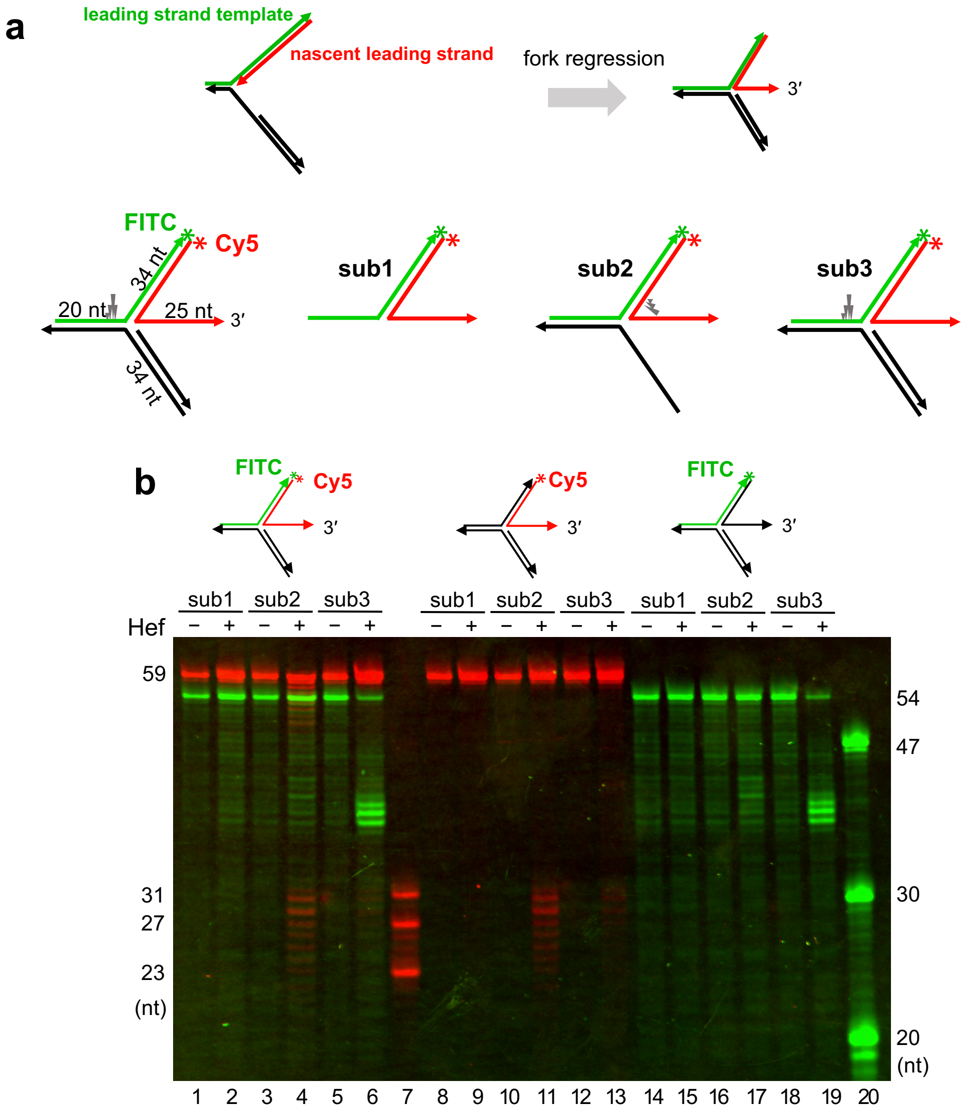

Supplementary Figure S5. Hef specifically cleaves the leading strand template. (**a**) The structures of sub1, sub2, and sub3 are shown. The cleavage sites in each substrate are indicated by arrowheads, with sizes representing the cutting efficiency. (**b**) Hef (5 nM as the dimer) was incubated with 50 nM of each substrate at 50° C for 10 min, and the products were analyzed by 8 M urea-15% PAGE. Lanes 7 and 20 show the size markers (lane 7: 31, 27, and 23 nt of Cy5-labeled DNA; lane 20: 47, 30, and 20 nt of FITC-labeled DNA). The template strand and nascent leading strand were labeled with FITC and Cy5, respectively, on the three types of DNAs, sub1, sub2, and sub3 (lanes 1-6). These DNAs were labeled at only nascent leading strand (lane 8-13), and at only template strand (14-19). The each labeled structure of sub3 was shown on the top of the panel as a representative.

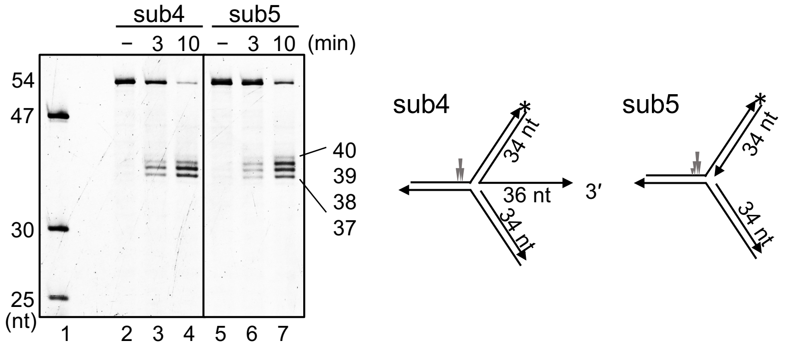

Supplementary Figure S6. Hef cleaves the leading strand template with the same efficiency, with or without the ssDNA region in the nascent leading strand. Hef (5 nM as the dimer) was incubated with 50 nM of each substrate at 50° C for 10 min, and the products were analyzed by 8 M urea-15% PAGE. Lane 1 shows the size markers (47, 30, and 25 nt). The “sub4” and “sub5” indicate the forks in which the leading strand is regressed to generate the 3′ overhanging ssDNA and the fork with no gap, respectively. The sites in the fork-structured DNA that are cleaved by Hef are indicated by the arrowheads. The size of the arrowhead represents the cleavage efficiency.

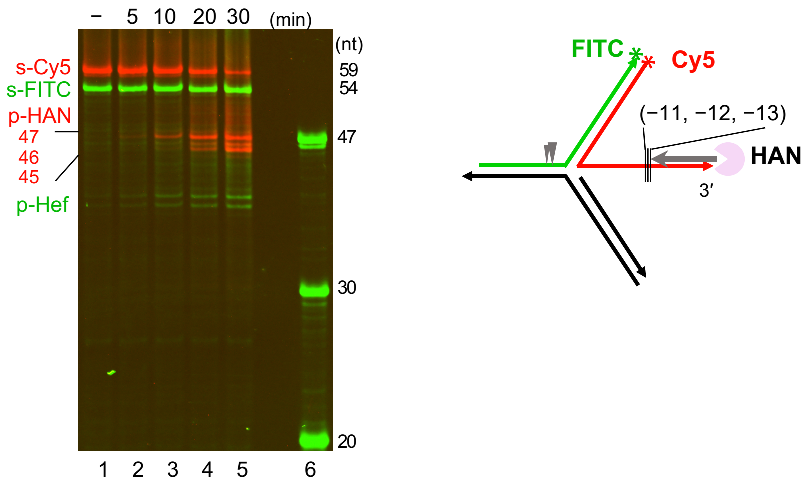

Supplementary Figure S7. The reaction products in Figure 4b were analyzed by 8 M urea-15% PAGE with higher mobility. HAN cleaves the 3′ overhanging ssDNA and leaves a 11–13 nt long ssDNA from the junction. Lane 6 shows the size markers (47, 30, and 20 nt). The band assignments are indicated on the side of the panel: s, substrates (s-Cy5, s-FITC); p, cleavage products by HAN (p-HAN, Cy5-labeled DNA) and Hef (p-Hef, FITC-labeled DNA).

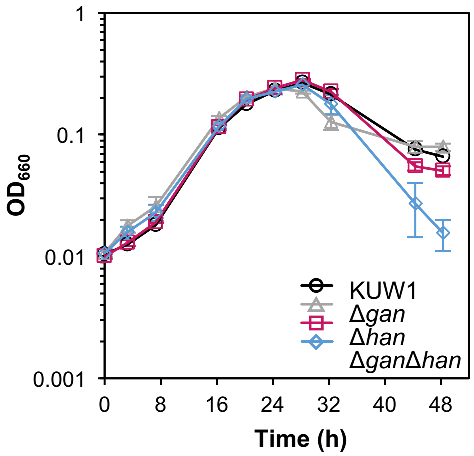

Supplementary Figure S8. Growth curves of the *T. kodakarensis* parental strain (KUW1), and the Δ*gan*, Δ*han*, and Δ*gan*Δ*han* strains at 70° C in ASW-YT-Pr medium.
